# Long range deuterium isotope effects on ^13^C NMR chemical shifts of 2-alkanones in CD_3_OD solutions of imidazolium acetate ionic liquids†‡

**DOI:** 10.1039/d1ra07232c

**Published:** 2021-12-07

**Authors:** Astghik A. Shahkhatuni, Aleksan G. Shahkhatuni, Arpine S. Harutyunyan

**Affiliations:** Scientific Technological Center of Organic and Pharmaceutical Chemistry of NAS RA 26 Azatutyan Yerevan 0014 Armenia astriksh@gmail.com

## Abstract

Deuterium isotope substitution in one part of a molecule could produce a significant effect on chemical shifts of neighbouring nuclei as well as on nuclei, located far from the site of replacement. To estimate how far this influence could extend the reaction of proton–deuterium exchange of several 2-alkanones in deuterated methanol solutions of 1-methyl 3-ethyl imidazolium acetate ionic liquid (IL) was studied in detail using ^13^C NMR spectroscopy. Deuteration occurs in alkyl groups of 2-alkanones neighboring the ketonic group *via* keto–enol tautomerization catalyzed by IL. In the course of the reaction, various isotopomers with various deuteration levels are formed, among which a dynamic equilibrium is established. The number of substituted deuterons affects not only the multiplicity and chemical shifts of directly bonded carbon, but carbons in the groups further along the alkyl chain. Moreover, the latter groups better indicate the level and site of substitution.

## Abstract 2

Long range deuterium isotope effects on the carbon spectral pattern in 2-hexanone reveal the existence and distribution of H/D isotopomers.

## Graphical abstract

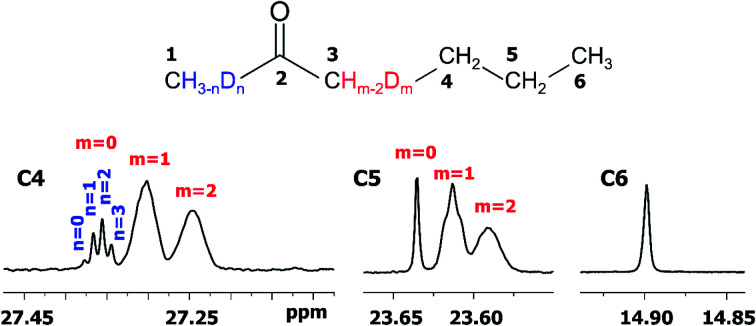

## Introduction

Deuterium isotope effects (DIE) are an effective tool for studying the structure and dynamics of organic and biological molecules,^1–3^ and for drug design.^4^ They are very useful for spectral interpretation and for structural elucidation in NMR spectroscopy as well. In such studies mainly DIE on ^1^H and ^13^C spectra over two bonds are used, but no less interesting is the transmission of the effects *via* three or more bonds. Moreover, it is known that DIE can be transmitted across hydrogen bonds.^5–7^

The wide use of DIE is limited by the difficultness and costliness of deuterium labelling.

However, H/D exchange reactions occurring in solutions of imidazolium ionic liquids (IL) in deuterated solvents containing exchangeable deuterons (like chloroform-d, methanol-d_6_ or heavy water) provide a good opportunity for such studies.^8^ Especially attractive is the simultaneous existence of large set of different isotopomers in the reaction mixture and variation of this set over the time.

Recently we have shown such possibility on an example of the simplest ketonic compound – acetone. We have observed DIE during the deuteration of acetone due to the exchange reaction *via* keto–enol tautomerization, occurring in heavy water environment only in the existence of IL served as a catalyst.^8^

DIE in ^13^C NMR spectra have been first reported in 1967 only for carbonyl carbon of acetone-d_6_.^9^ Later DIE were studied thoroughly in ten compounds by Sergeyev *et al.*^10^ In all cases the upfield shift approximately 0.1–0.3 ppm per one substituted deuterium was observed over two bonds, and 0.02–0.04 ppm over three bonds. The suggestion about additivity of DIE on carbons *versus* number of substituted deuteriums was made, which was later confirmed for all studied acyl derivatives.^11^

Long range DIE is observed in conjugated p-electron molecules many bonds away from the isotopic substitution site (up to twelve),^12^ or in molecules with intramolecular hydrogen bonding.^13–15^

Here we report the study of long range DIE on ^1^H and ^13^C{H} spectra of several simple 2-alkanones, dissolved in mixture of [C_2_mim][OAc] ILs and methanol-d_4_ with the aim to determine the influence of deuterium substitution on NMR chemical shifts over two or more bonds from the substitution site.

## Experimental

Seven 2-alkanones (acetone, methyl ethyl ketone, methyl propyl ketone, methyl isopropyl ketone, methyl butyl ketone, methyl *tert*-butyl ketone, methyl hexyl ketone), and acetophenone studied here were dissolved in methanol-d_4_ solution of 1-ethyl-3-methylimidazolium acetate [C_2_mim][OAc] ionic liquid and monitored for a few months, although the equilibrium was usually reached in a few weeks. The structures and abbreviations used for ease of following throughout the text are given in Table 1. ^1^H and ^13^C{H} NMR spectra were registered periodically to detect various level and number of isotopomers. Samples were stored at room temperature between measurements.

**Table 1:** The structure and abbreviations of studied compounds

Compound	Structure	Abbreviation
1-Ethyl-3-methyl-imidazolium acetate	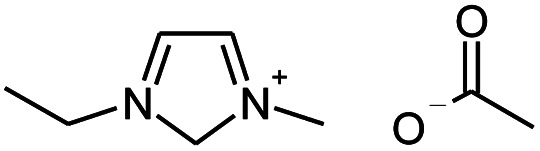	[C_2_mim][OAc]
Acetone	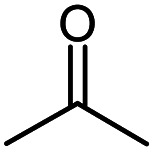	Acetone
Methyl ethyl ketone	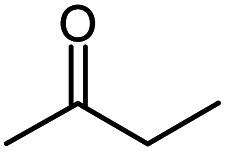	MEK
Methyl propyl ketone	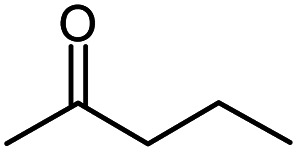	MPK
Methyl isopropyl ketone	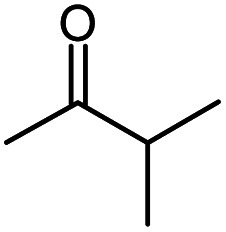	MiPK
Methyl butyl ketone	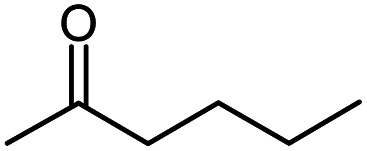	MBK
Methyl *tert*-Butyl ketone	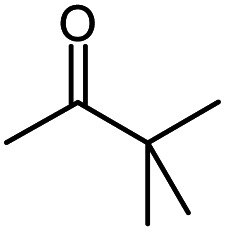	MtBK
Methyl hexyl ketone	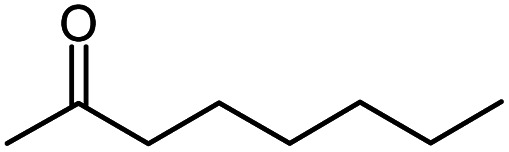	MHK
Acetophenone	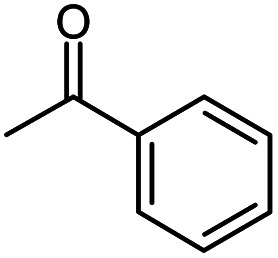	MPhK

All used ketones are commercially available and were purified before use. Methanol-d_4_ was purchased from Cambridge Isotope Laboratories. [C_2_mim][OAc] IL was obtained from Alfa Aesar. Composition of all samples is 1 : 1 v/v methanol/IL with 4.0% v/v of ketone in the mixture.

Standard ^1^H and inverse gated ^13^C{H} NMR spectra of samples were acquired at 303 K on 400 MHz Bruker AVANCE NEO spectrometer equipped with a temperature controlled Smart probe. The number of scans for ^13^C{H} were 8–16 K, acquisition time of about 5 s and delay time 5 s. Chemical shifts in spectra were referenced by signal of methanol-d_4_ and taken 49.15 ppm for ^13^C spectra and 3.31 ppm for ^1^H spectra. In the ^13^C{H} spectra the linewidths of solute signals, undistorted from long range interactions and without postprocessing, are about 0.2–0.4 Hz with spectral resolution of 0.09 Hz. MestReNova software was used to process spectra. The most relevant spectra are included in ESI.‡

## Results and discussion

Earlier we have studied thoroughly the proton–deuterium exchange of acetone *via* keto–enol tautomeric equilibrium reactions in heavy water catalyzed by imidazolium-based ILs.^8^ As we have shown, the effectiveness of H/D exchange in various imidazolium-based IL mixtures is different, and both the nature of anion and the alkyl chain length can play crucial role. During this study we reconfirmed, that in particular, IL with acetate anion and shorter alkyl chain are more effective catalysts. Thus, below only the samples with [C_2_mim][OAc] IL as a catalyst are considered.

Comparison of H/D exchange processes of several simple ketones in different solvents showed that the reactions in methanol-d_4_/IL occur faster than, for instance, in chloroform-d or heavy water. However, at the same volumes of CD_3_OD and D_2_O in the IL mixtures, due to the higher number of exchangeable deuterons in heavy water, the deuteration level in the latter is considerably higher over extended periods. The advantage of using CD_3_OD in this study is the possibility to have most of deuterium isotopomers in one sample simultaneously, which is very important to reveal isotope effects on chemical shifts.

Thus, here we reveal the peculiarities of DIE on chemical shifts of directly connected carbons and carbons distanced by several bonds in 2-alkanones using [C_2_mim][OAc]/CD_3_OD mixtures at the same conditions. The deuteration occurs in directly neighbouring groups in both sides of carbonyl carbon. The H/D exchange reaction in ketones in this solution starts immediately and a set of isotopomers with various level of deuteration is generated with varying relative amount over the time. The process of deuterium substitution continues until the equilibrium with the reverse process of D/H exchange is established.

The ^13^C{H} NMR spectral pattern of such mixtures during the reaction is very informative and illustrative. DIE on ^13^C chemical shifts are described as the difference between chemical shifts of the non-deuterated and deuterated molecules:^*k*^Δ^13^C(D) = *δ*^13^C(H) − *δ*^13^C(D)where *k* is the number of bonds between the site of deuteration and the observed carbon. In general, various structural factors such as hybridization, conjugation, torsional angle, resonance, *etc.* can affect the magnitude and the sign of isotope effects.^1^ However, DIE mainly have rotational-vibrational origin and arise due to anharmonicity of the potential curve of the X–H(D) bond.^12,16^

Below we will discuss peculiarities of NMR parameters of carbons at various positions in 2-alkanones obtained from ^13^C{H} NMR spectra as well as NMR information available from ^1^H spectra. The numbering of carbons is done following IUPAC recommendations.

### DIE on C1 carbon

**Fig. 1:**
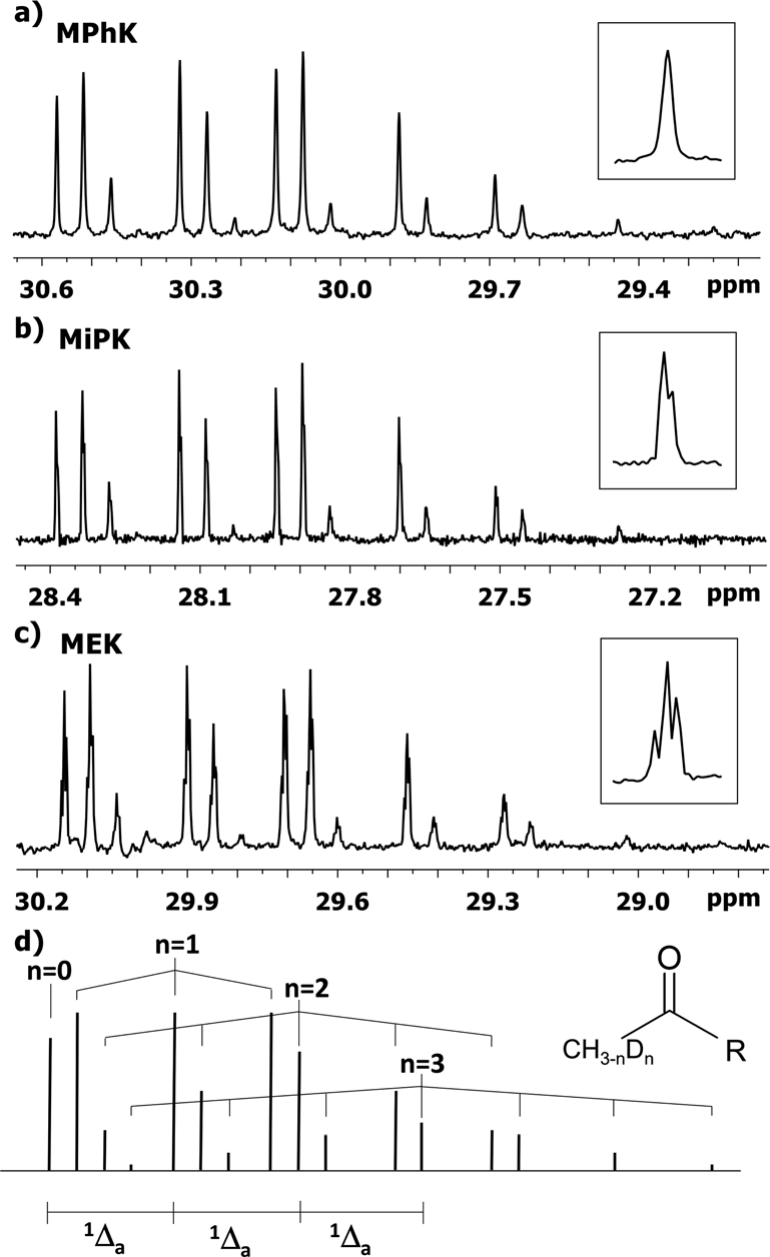
^13^C{H} spectral pattern of C1 carbon of (a) MPhK, (b) MiPK, and (c) MEK in [C_2_mim][OAc]/CD_3_OD after three, seven, and six weeks of H/D exchange reaction, respectively. The typical patterns of all lines in corresponding spectra are presented in the expanded windows. (d) The schematic representation of isotopomers with various number of deuteriums (*n* = 0, 1, 2, 3) at C1 carbon and additivity of chemical shift ^1^Δ_a_(C1).

The behaviour of ^13^C chemical shifts of C1 carbons in CH_3_ group is similar for all studied 2-alkanones (Fig. 1) and was demonstrated in details for acetone.^8^ The H/D substitution occurs in methyl group *via* the keto–enol tautomerization and four isotopomers with *n* = 0, 1, 2, and 3 deuterons are generated. Due to the large value of *J*_CD_ coupling constant and different chemical shifts of methyl carbon in CH_3_, CH_2_D, CHD_2_, and CD_3_ containing isotopomers, the corresponding signals observed in ^13^C{H} NMR spectra are well resolved. Spectral patterns of these groups are characteristic and appear as an easily recognizable singlet, triplet (1 : 1 : 1), quintet (1 : 2 : 3 : 2 : 1), and septet (1 : 3 : 6 : 7 : 6 : 3 : 1), correspondingly (Fig. 1d).

The C1 spectral pattern for MtBK and MPhK is the simplest from studied ketones, since there is only one possible deuterium substitution site (Fig. 1a).

As in case of acetone,^8^ there is significant (0.24–0.25 ppm) upfield shift for each consequent isotopomer for all studied ketones.

Although there is a similar pattern for C1 in all studied 2-alkanones, however for non-symmetric 2-alkanones there is superimposition of several slightly shifted similar multiplets, corresponding to possible deuterated groups (CH or CH_2_) at the C3 carbon. The characteristic pattern for each line is shown in the expanded regions for different ketonic compounds (Fig. 1a–c). Since for MiPK there are only two possibilities (CH and CD), there are only two lines with various intensities reflecting the amount of corresponding isotopomers due to the deuteration at C3 site (Fig. 1b).

However, the signals are not well resolved and only allow rough estimation. For MEK there are three possibilities (CH_2_, CHD, CD_2_), and we can see three lines (Fig. 1c). The chemical shift differences are small (less than 0.005 ppm), and are again shifted upfield and linearly distanced.

The following expression describes the final chemical shift of C1 carbon of any possible H/D isotopomer for linear 2-alkanones. The expression is the linear combination of upfield shields of C1 carbon due to deuteration at C1 and/or C3 sites multiplied by the number of deuteriums at each substitution site:

1 ^1^Δ(C1)(CH_3−*n*_D_*n*_COCH_2−*m*_D_*m*_(CH_2_)_*p*_CH_3_) = *n*^1^Δ_a_(C1) +*m*^3^Δ_b_(C1)

where ^1^Δ_a_(C1) and ^3^Δ_b_(C1) are chemical shift differences arising from only one deuterium substitution of protons directly connected with C1 and C3 carbons, correspondingly; *n* (0, 1, 2 or 3) and *m* (0, 1 or 2) are the number of substituted deuteriums at those sites; and *p* (0,1,2,4) is the number of methylene groups in studied 2-alkanone.

In case of MiPK, the slightly different expression (1) can be used replacing CH_2−*m*_ with CH_1−*m*_, where *m* = 0 or 1. For MtBK and MPhK only the first term of expression (1) should be used.

The ^1^*J*_CD_ is the same for all studied 2-alkanones and equals to 19.5 Hz.

### DIE on C2 carbonyl carbon

**Fig. 2:**
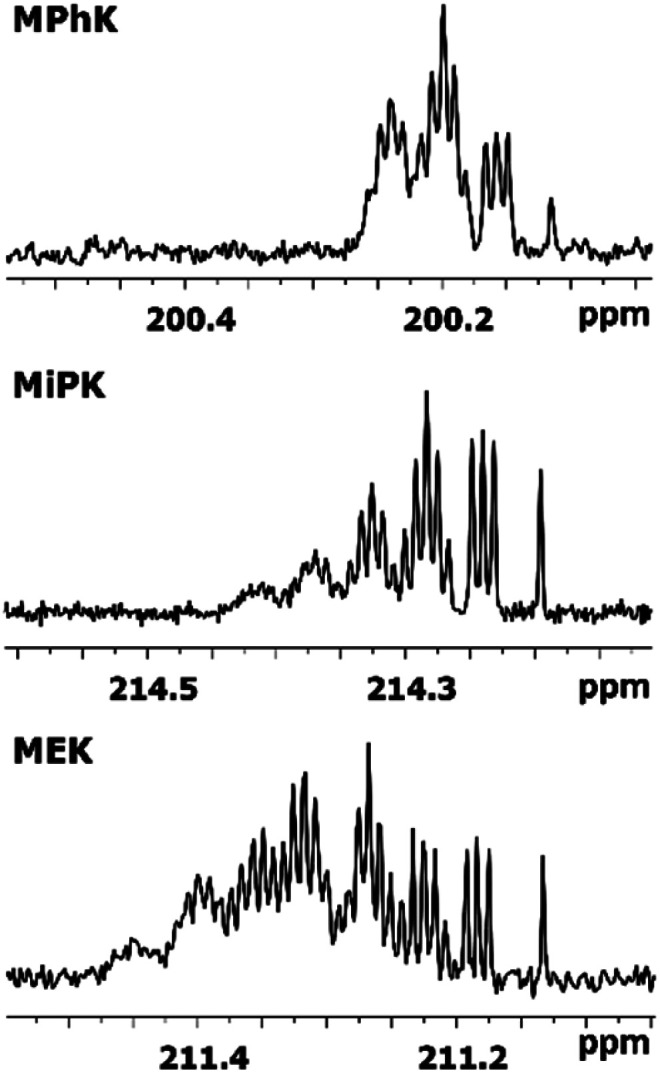
^13^C{H} spectral pattern of C2 carbon in MPhK, MiPK, and MEK in [C_2_mim][OAc]/CD_3_OD after three, seven and six weeks of H/D exchange reaction, respectively.

The C2 spectral pattern for MPhK is shown on Fig. 2. The singlet for CH_3_, the triplet for CH_2_D, the quintet for CHD_2_ and the septet for CD_3_ groups for corresponding isotopomers can be registered with the linear downfield chemical shift of 0.042 ppm for all isotopomers.

In case of acetone the spectral pattern of carbonyl carbon is also very informative and unambiguously shows the deuteration level. It consists of 7 superimposed multiplets, corresponding to isotopomers with various numbers of deuteriums in the molecule (from 0 to 6) with the same downfield chemical shift of −0.0587 ppm.^8^

However, for non-symmetrical 2-alkanones, the DIE effects depend on the substitution site (C1 or C3), leading to differentiation of multiplets corresponding to different isotopomers with the same number of deuteriums in the molecule. As a result, C2 carbonyl carbon spectral pattern is much more complex (Fig. 2), with combination of two non-equal linear downfield chemical shifts for each site of substitution.

In other words, the effect of deuteration at C1 site on the chemical shifts differs from the one at C3 site.

In particular, in case of MiPK, the effect of CD group at C3 carbon makes the spectral pattern different from MPhK. The analysis showed that there is downfield shift of −0.085 ppm and −0.045 ppm caused by CD group and each substituted deuterium in methyl group at C1, correspondingly.

For MEK and other linear 2-alkanones, the spectral pattern of C2 is more complicated, nonetheless it is easily interpretable by linear combination of two linear downfield effects on chemical shifts from deuterated groups at C1 and C3 sites (Fig. 2):

2 ^2^Δ(C2)(CH_3−*n*_D_*n*_COCH_2−*m*_D_*m*_(CH_2_)_*p*_CH_3_) = *n*^2^Δ_a_(C2) + *m*^2^Δ_b_(C2)

**Fig. 3:**
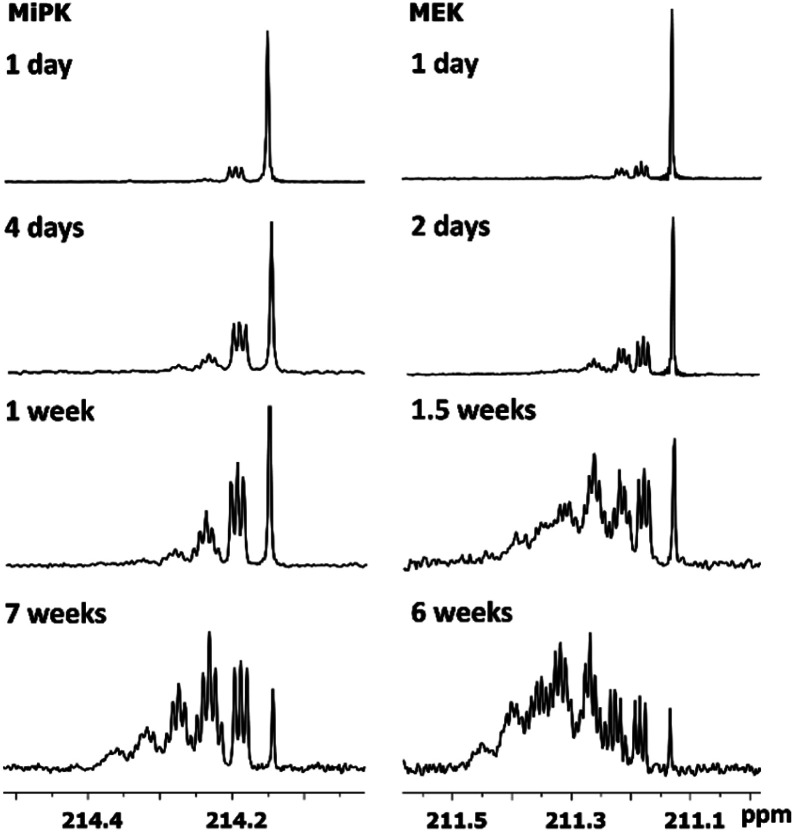
Change of ^13^C{H} spectral pattern of carbonyl C2 of MiPK and MEK due to consequent deuteration monitored over the time.

The consequent deuteration over the time allows to monitor the difference of C2 carbon pattern of MiPK *versus* MEK (Fig. 3). For MiPK the triplet responsible for CH_2_DCOCH(CH_3_)_2_ isotopomer, then the quintet for CHD_2_COCH(CH_3_)_2_ appear first. While for MEK, the triplet from CH_2_DCOCH_2_CH_3_ is followed by the triplet from CH_3_COCHDCH_3_ isotopomer. The chemical shift variations are different in this case and can be clearly measured. The higher rate of deuteration leads to overlapping of quintet from CHD_2_COCH_2_CH_3_ with already existing triplet from CH_3_COCHDCH_3_ (Fig. 3).

It should be noted, that unlike chemical shifts, the ^2^*J*_CD_ of C2 for all 2-alkanones is the same and equals to 0.85 Hz irrespective of deuteration site and alkyl chain length.

### DIE on C3 carbon

**Fig. 4:**
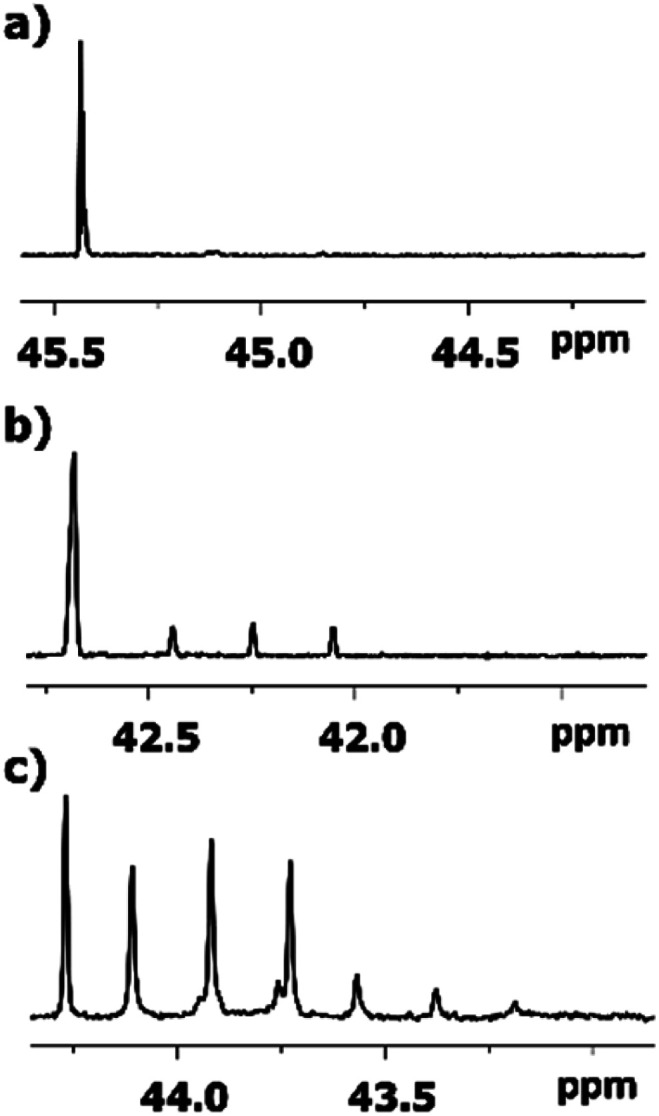
^13^C{H} NMR signals of C3 carbon in various 2-alkanones: (a) quaternary carbon in MtBK (b) CH group in MiPK; (c) and CH_2_ group in MBK after 3, 7, and 6 weeks, respectively.

As expected, the deuteration occurs at both sides of carbonyl carbon of 2-alkanones, albeit with various spectral pattern and rate. As opposed to the singlet line in MtBK, in MiPK for C3 carbon the singlet line of non-deuterated CH group, and the triplet for CD, will appear consecutively (Fig. 4). Obviously, for MEK, over the time along with corresponding singlet and triplet, the quintet due to CD_2_ containing isotopomers will appear as well (Fig. 4c). The similar although larger upfield shift ^1^Δ_b_(C3) of about 0.33–0.44 ppm is registered for linear 2-alkanones.

The influence of deuterated C1 methyl group in MtBK can be clearly seen as a combination of 4 lines responsible to four possible isotopomers at C1 site with ^3^Δ_a_(C3) about 0.007 ppm. However, for all other 2-alkanones, the multiplets of all signals of C3 carbons, corresponding to various isotopomers deuterated at C1 site, are not resolved, with estimated from linewidths chemical shift differences ^3^Δ_a_(C3) being less than 0.002 ppm.

The ^1^*J*_CD_ of C3 carbon slightly varies depending on alkyl substituent (19.1–19.5 Hz).

### DIE on carbons further in the chain of 2-alkanones

It is known, that DIE on chemical shifts of carbons can be observed over two or more bonds from the deuteration site in the longer chain of alkanes.^1,15^ Indeed, in our studied 2-alkanones the observed chemical shifts of alkyl carbons after C3 carbon along the chain are perturbed by deuteration at C1 and/or C3 sites.

**Fig. 5:**
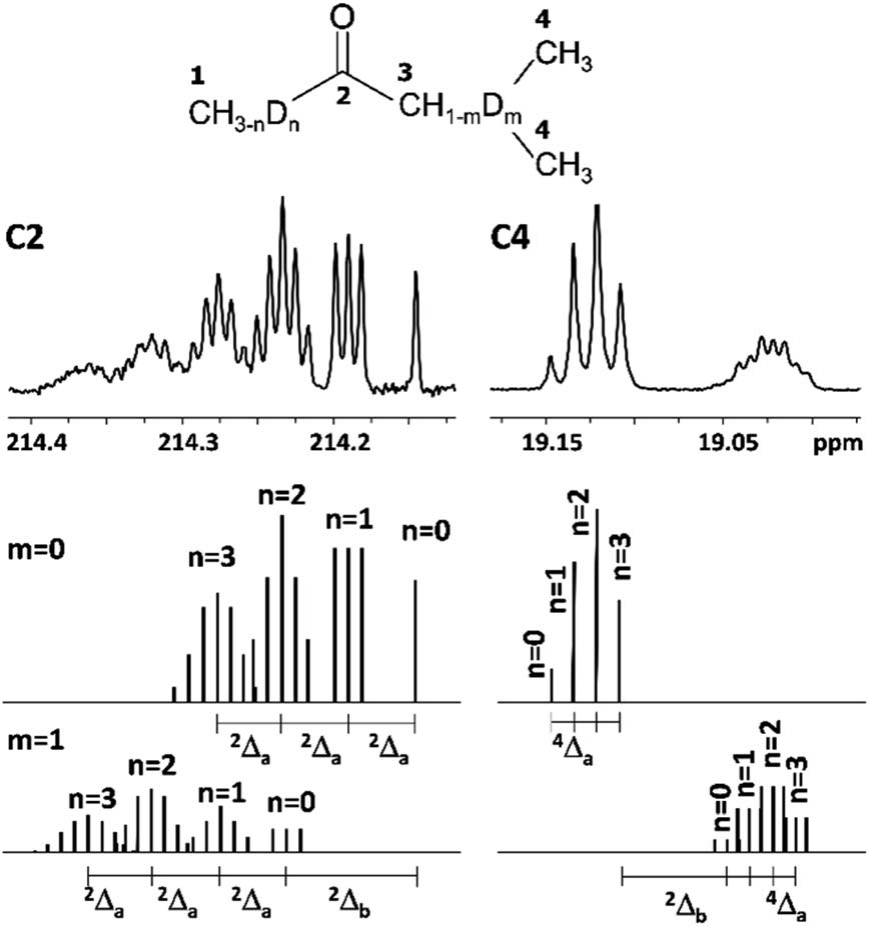
Additivity of DIE on C2 and C4 carbons of MiPK, where *n* = 0, 1, 2, 3 and *m* = 0, 1 indicate the number of deuteriums in methyl group and CH group at C1 and C3 sites, respectively.

As we have already discussed for carbonyl carbon C2, which is non-directly connected to substituted deuteriums, the chemical shifts are affected by both C1 and C3 deuteration sites. Furthermore, the resulting chemical shifts are linear combination of both downfield effects ^2^Δ_a_(C2) and ^2^Δ_b_(C2) (eqn (2)), which is drawn schematically for MiPK on Fig. 5. The real spectrum is shown on the upper part of Fig. 5, and below it is the schematic representation of isotopomers with non-deuterated (*m* = 0) and deuterated (*m* = 1) C3 site.

Analogously, C4 carbon region of ^13^C{H} spectrum in MiPK consists from two multiplets corresponding to nondeuterated (*m* = 0) and deuterated (*m* = 1) isotopomers at C3 carbon, separated by the significant upfield chemical shift of ^2^Δ_b_(C4) = 0.1 ppm (Fig. 5). For isotopomers with nondeuterated CH group (*m* = 0) there are 4 possibilities of deuteration of methyl group at C1 site, and correspondingly 4 singlets arise responsible for 4 isotopomers (*n* = 0, 1, 2, 3) in downfield part of C4 region. If CH group at C3 site is deuterated (*m* = 1), then again 4 possibilities will arise because of *n* = 0, 1, 2 or 3 substituted deuterons at C1 site, and accordingly in the spectra the complex signal from all these isotopomers with corresponding multiplicities (four triplets with ^2^*J*_CD_ = 0.65 Hz in case of MiPK) is seen. The registered upfield chemical shift ^4^Δ_a_(C4) is about 0.01 ppm.

**Fig. 6:**
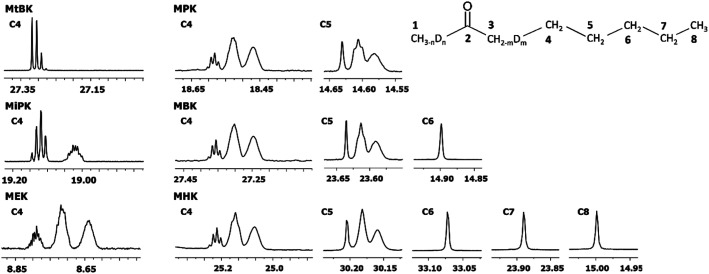
^13^C{H} spectral pattern of alkyl chain in 2-alkanones (in ppm). The numbering of carbons is shown on an example of MHK (*n* = 0, 1, 2, 3; *m* = 0, 1, 2).

In Fig. 6 DIE on the rest of the carbons along the alkyl chain of 2-alkanones are shown. For linear 2-alkanones C4 carbon spectral pattern consists from three distinct parts, responsible for *m* = 0, 1, 2 isotopomers (Fig. 6), two of which are unresolved groups of multiplets, and the downfield signal is a combination of singlets with *n* = 0, 1, 2, 3 substituted deuteriums at C1 site. The ^2^Δ_b_(C4) for MEK is found to be 0.075 ppm, and 0.055 ppm for other 2-alkanones per each substituted deuterium at C3 site. ^4^Δ_a_(C4) is about 0.01 ppm. The expression for chemical shifts similar to (eqn (2)) could be used for C4.

The ^2^*J*_CD_ of C4 for MEK is 0.65 Hz, and is about 0.5 Hz or less for other 2-alkanones. It should be noted, that for MEK the downfield signal for nondeuterated isotopomers (*m* = 0) is too complex possibly as a result of stronger isotope effects of deuteration at C1 site leading to larger splittings due to long range spin–spin interactions and overlapping of appropriate multiplets.

Similar to C4 carbon pattern, the C5 carbon region is a combination of three distinct multiplets with *m* = 0, 1, 2, and ^3^Δ_b_(C5) is about 0.02 ppm. The effect of deuterated CH_3_ group at C1 site is not detectable on C5 and further carbons along the chain. The effect of deuteration of CH_2_ group at C3 carbon affects visibly only up to C5 carbons.

The data for chemical shift differences for all studied 2-alkanones is summarized in the Table 2. Signs and values of DIE in 2-alkanones are in good agreement with those, observed by others for alkyl groups,^12–15^ except experimental data for ^4^Δ of alkanes,^15^ for which negative DIE (−5.4 ± 0.5 ppb) was detected.

Vicinal DIE on H5 is less than 10 ppb. The corresponding data is presented in the Table 3.

**Table 2:** Additive linear changes of ^13^C chemical shifts of 2-alkanones *versus* H/D substitution site (in ppb). Positive values denote upfield shift

	C1		C2		C3		C4		C5	
	^1^Δ_a_	^3^Δ_b_	^2^Δ_a_	^2^Δ_b_	^1^Δ_b_	^3^Δ_a_	^2^Δ_b_	^4^Δ_a_	^3^Δ_b_	^5^Δ_a_
MtBK	237.8	—	—	—	—	6.6	—	13.5	—	—
MiPK	246.5	4.0	−44.7	−85.5	437.3	<2	99.3	12.9	—	—
MEK	245.5	5.0	−49.7	−84.5	334.9	<2	74.5	10.4	—	—
MPK	247.3	2.0	−51.7	−84.5	357.8	<2	54.7	10.4	24.8	0
MBK	247.5	3.5	−51.7	−84.5	350.8	<2	54.7	10.4	21.9	0
MHK	247.5	3.0	−51.7	−84.5	352.8	<2	54.7	10.9	22.9	0
Acetone	246.5		−55.2							
MPhK	240.5	—	−41.7	—						

**Table 3:** Additive linear changes of ^1^H chemical shifts of 2-alkanones *versus* H/D substitution site (in ppb). Positive values denote upfield shift

	H1		H3		H4
	^2^Δ_a_	^4^Δ_b_	^2^Δ_b_	^4^Δ_a_	^3^Δ_b_
MiPK	16.5	1.0	—	<0.75	9.25
MEK	16.5	1.0	27.6	1.38	6.5
MPK	16.7	1.0	27.5	1.25	
MBK	16.5	1.0	27.5	1.0	
MHK	16.7	1.0	27.5	1.0	

Both ^1^H and ^13^C{H} NMR spectra provide information about isotopomers in the mixture, however ^13^C{H} spectra is preferable, because of overlapping of some ^1^H signals, especially corresponding to alkyl groups of longer chains, and overall, smaller changes of chemical shifts and additional *J*_HH_ spin–spin coupling constants. Geminal DIE on the H1 is 16.5–16.7 ppb and about 27.5 ppb for H3. DIE over four bonds is about 1 ppb.

The proton-deuterium spin–spin coupling constants are small. For H1 the ^2^*J*_HD_ = 2.2 Hz, for H4 ^3^*J*_HD_ is about 1 Hz, and for H3 the ^2^*J*_HD_ is about 2.6 Hz and ^4^*J*_HD_ = 0.4 Hz.

Summarizing, the separation of NMR signals of various isotopomers due to DIE on chemical shifts allows to easily distinguish the site and level of deuterium substitution. Each carbon is influenced differently and its NMR pattern carries unique information. Combining information from various carbons the site of deuteration in the ketonic compound and its corresponding level can be determined.

In all cases a positive effect on ^13^C chemical shifts (observed shifts of deuterated compounds are upfield) of alkyl groups was determined, while DIE on carbonyl carbon is negative (downfield shifts). Shift values are linearly correlated on the number of substituted deuteriums. Thus, in 2-alkanones the H/D substitution influences the ^3^*J*_CD_ carbon-deuterium spin–spin couplings across at least 3 bonds and the chemical shifts up to 4 bonds.

## Conclusions

H/D substitution reactions in 2-alkanones in IL/methanol-d_4_ mixtures produce a set of isotopomers with various number of deuteriums in molecules at C1 and C3 carbons, allowing to study deuterium isotope effects on ^13^C carbon NMR chemical shifts. Moreover, the deuterium substitution affects chemical shifts and multiplicity in spectral pattern of carbons up to four bonds away from the substitution site. It was determined that DIE on all affected chemical shifts are the sum of influences of both substitution sites and are linear *versus* the number of substituted deuteriums at each site. Particularly, the ^13^C chemical shift differences in carbons directly bonded with substitution site can reach 0.24–0.25 ppm for C1 carbon and 0.33–0.44 ppm for C3 carbon in 2-alkanones. Long range DIE on chemical shifts of carbons vary by 0.01–0.02 ppm per substituted deuterium. It must be noted, that signal patterns of the carbons further along the chain are more informative and easier interpretable tool for the visualisation of the distribution of isotopomers arising during the H/D exchange reaction ongoing with 2-alkanones.

## Conflicts of interest

There are no conflicts to declare.

## Supplementary Material

RA-011-D1RA07232C-s001

## References

[cit1] Hansen P. E. (1988). Prog. Nucl. Magn. Reson. Spectrosc..

[cit2] Berger S. , Van Etten R. L., Risley J. M. and Sergeyev N. M., NMR Basic Principles and Progress, Publisher, Springer Berlin Heidelberg, 1990, vol. 22, p. 172

[cit3] Dziembowska T., Hansen P. E., Rozwadowski Z. (2004). Prog. Nucl. Magn. Reson. Spectrosc..

[cit4] Hansen P. E. (2021). Molecules.

[cit5] Schah-Mohammedi P., Shenderovich I. G., Detering C., Limbach H.-H., Tolstoy P. M., Smirnov S. N., Denisov G. S., Golubev N. S. (2000). J. Am. Chem. Soc..

[cit6] Remsing R. C., Wildin J. L., Rapp A. L., Moyna G. (2007). J. Phys. Chem..

[cit7] Hansen P. E. (2015). Molecules.

[cit8] Shahkhatuni A. A., Shahkhatuni A. G., Mamyan S. S., Ananikov V. P., Harutyunyan A. S. (2020). RSC Adv..

[cit9] Maciel G. E., Paul D. E., Hofer D. C. (1967). J. Phys. Chem..

[cit10] Grishin Y. K., Sergeyev N. M., Ustynyuk Y. A. (1971). Mol. Phys..

[cit11] Hansen P. E., Nicolaisen F. M., Schaumburg K. (1986). J. Am. Chem. Soc..

[cit12] Novak P., Pičuljan K., Biljan T., Hrenar T., Cindrić M., Rubčić M., Meić Z. (2007). Croat. Chem. Acta.

[cit13] Hansen P., Kamounah F., Gryko D. (2013). Molecules.

[cit14] Buceta N. N., Della Védova C. O., Romanelli G. P., Autino J. C., Jios J. L. (2008). J. Mol. Struct..

[cit15] Wesener J. R., Moskau D., Günther H. (1985). J. Am. Chem. Soc..

[cit16] Gräfenstein J. (2019). J. Chem. Phys..

