# Supplementary material for: Long range deuterium isotope effects on 13C NMR chemical shifts of 2-alkanones in CD3OD solutions of imidazolium acetate ionic liquids
Source: RSC Adv. 2021 Dec 7;11(62):39051–7. doi: 10.1039/d1ra07232c (PMC9044476; doi:10.1039/d1ra07232c)

Electronic Supplementary Material (ESI) for RSC Advances.
This journal is © The Royal Society of Chemistry 2021

Supporting Information

Long range deuterium effects on ^{13}C NMR chemical shifts of 2-alkanones in CD_3OD solutions of imidazolium acetate ionic liquids.

Astghik A. Shahkhatuni, Aleksan G. Shahkhatuni, and Arpine S. Harutyunyan

^{13}C NMR spectra of ketones in $C_2mimOAc/CD_3OD$

1. Methyl Isopropyl Ketone
    a. after 1 day
    b. after 4 days
    c. after 6 days
    d. after 7 weeks
2. Methyl Ethyl Ketone
    a. after 1 day
    b. after 2 days
    c. after 11 days
    d. after 6 weeks
3. Methyl Propyl Ketone
4. Methyl Butyl Ketone
5. Methyl Hexyl Ketone
6. Methyl *Tert*-Butyl Ketone
7. Acetophenone
8. Acetone

^{1}H NMR spectra of ketones in $C_2mimOAc/CD_3OD$

1. Methyl Isopropyl Ketone in $C_2mimOAc/CD_3OD$
2. Methyl Ethyl Ketone in $C_2mimOAc/CD_3OD$
3. Methyl Propyl Ketone in $C_2mimOAc/CD_3OD$
4. Methyl Butyl Ketone in $C_2mimOAc/CD_3OD$
5. Methyl Hexyl Ketone in $C_2mimOAc/CD_3OD$

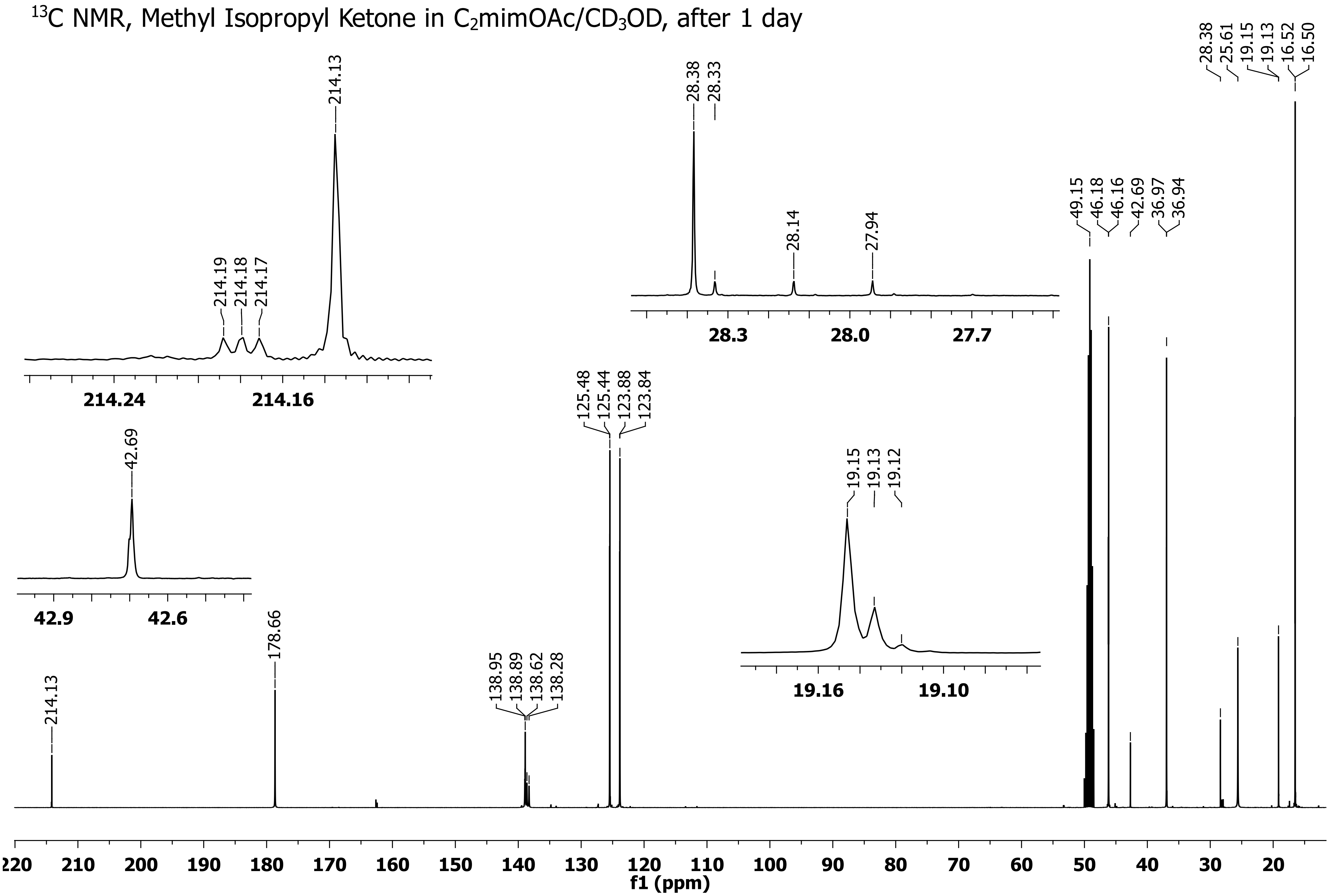

13C NMR, Methyl Isopropyl Ketone in C2mimOAc/CD3OD, after 1 day
214.13
214.19
214.18
214.17
214.24
214.16
28.38
28.33
28.14
27.94
28.3
28.0
27.7
49.15
46.18
46.16
42.69
36.97
36.94
28.38
25.61
19.15
19.13
16.52
16.50
125.48
125.44
123.88
123.84
42.69
42.9
42.6
19.15
19.13
19.12
19.16
19.10
178.66
138.95
138.89
138.62
138.28
220
210
200
190
180
170
160
150
140
130
120
110
100
90
80
70
60
50
40
30
20
f1 (ppm)

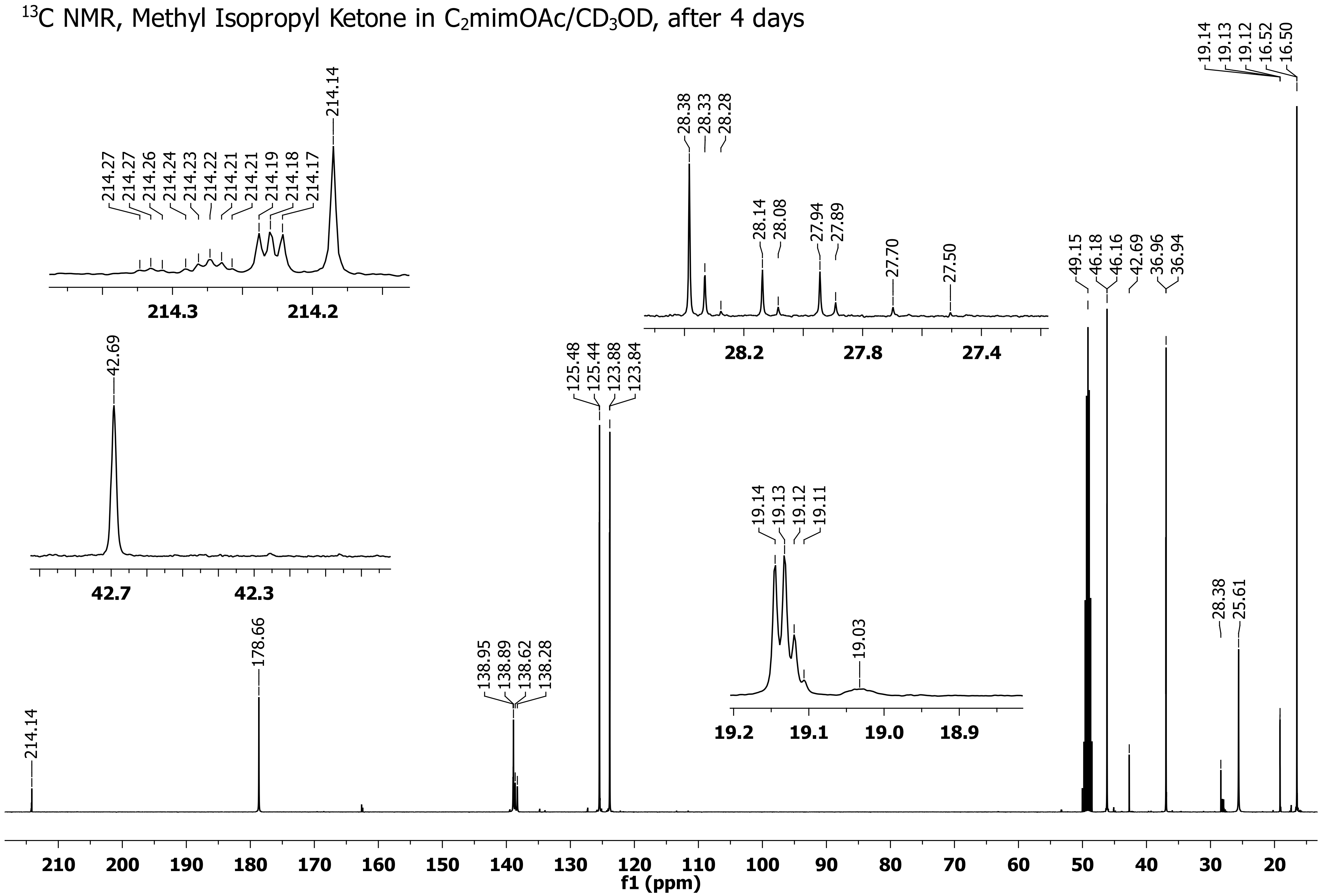

^{13}C NMR, Methyl Isopropyl Ketone in C_2mimOAc/CD_3OD, after 4 days
214.14
178.66
138.95
138.89
138.62
138.28
125.48
125.44
123.88
123.84
49.15
46.18
46.16
42.69
36.96
36.94
28.38
25.61
19.14
19.13
19.12
16.52
16.50
214.27
214.27
214.26
214.24
214.23
214.22
214.21
214.21
214.19
214.18
214.17
214.14
214.3
214.2
28.38
28.33
28.28
28.14
28.08
27.94
27.89
27.70
27.50
28.2
27.8
27.4
42.69
42.7
42.3
19.14
19.13
19.12
19.11
19.03
19.2
19.1
19.0
18.9
210
200
190
180
170
160
150
140
130
120
110
100
90
80
70
60
50
40
30
20
f1 (ppm)

^{13}C NMR, Methyl Isopropyl Ketone in $C_2mimOAc/CD_3OD$, after 6 days

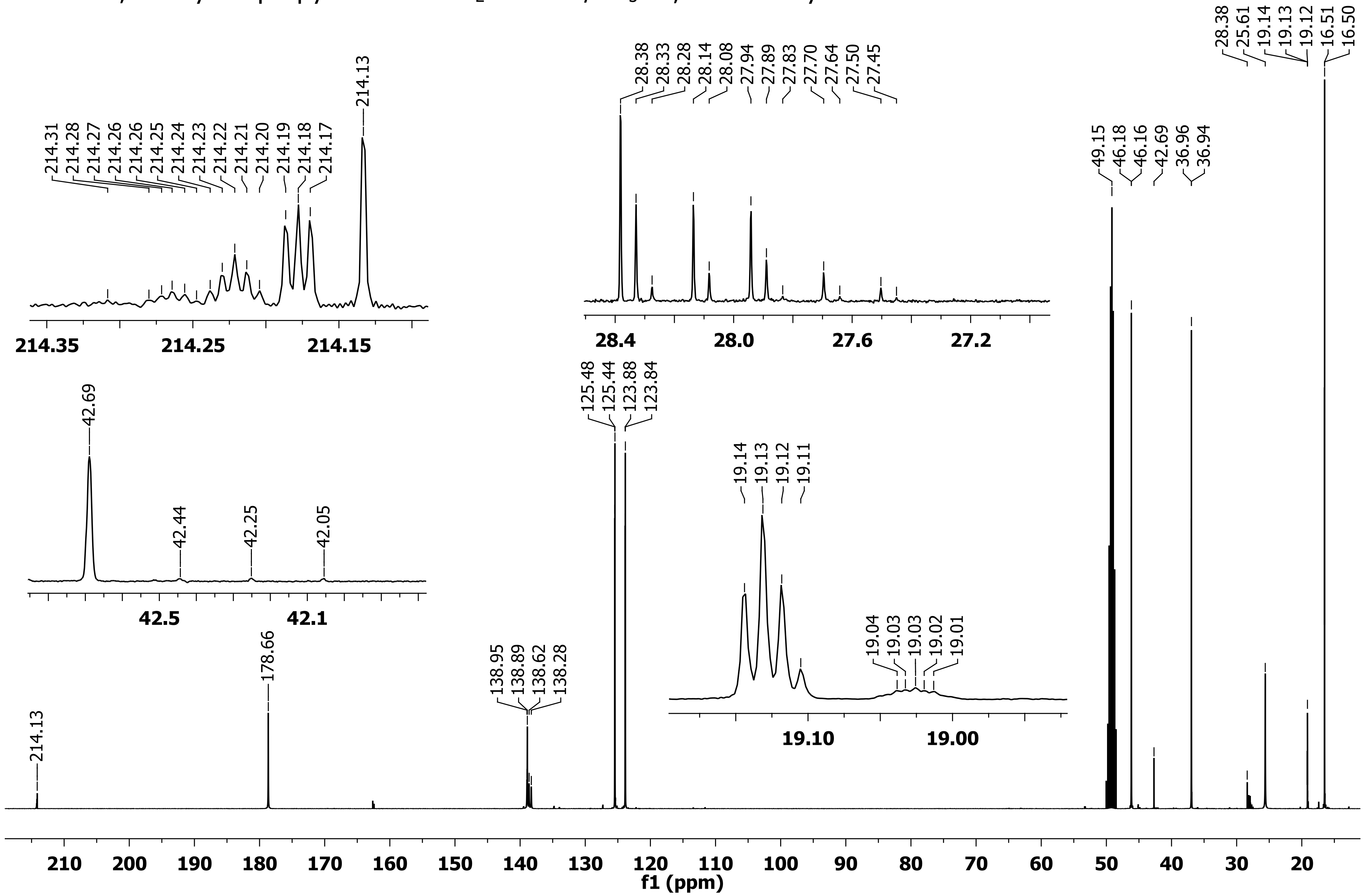

Methyl isopropyl ketone in C2mimOAC/CD3OD, after 7 weeks

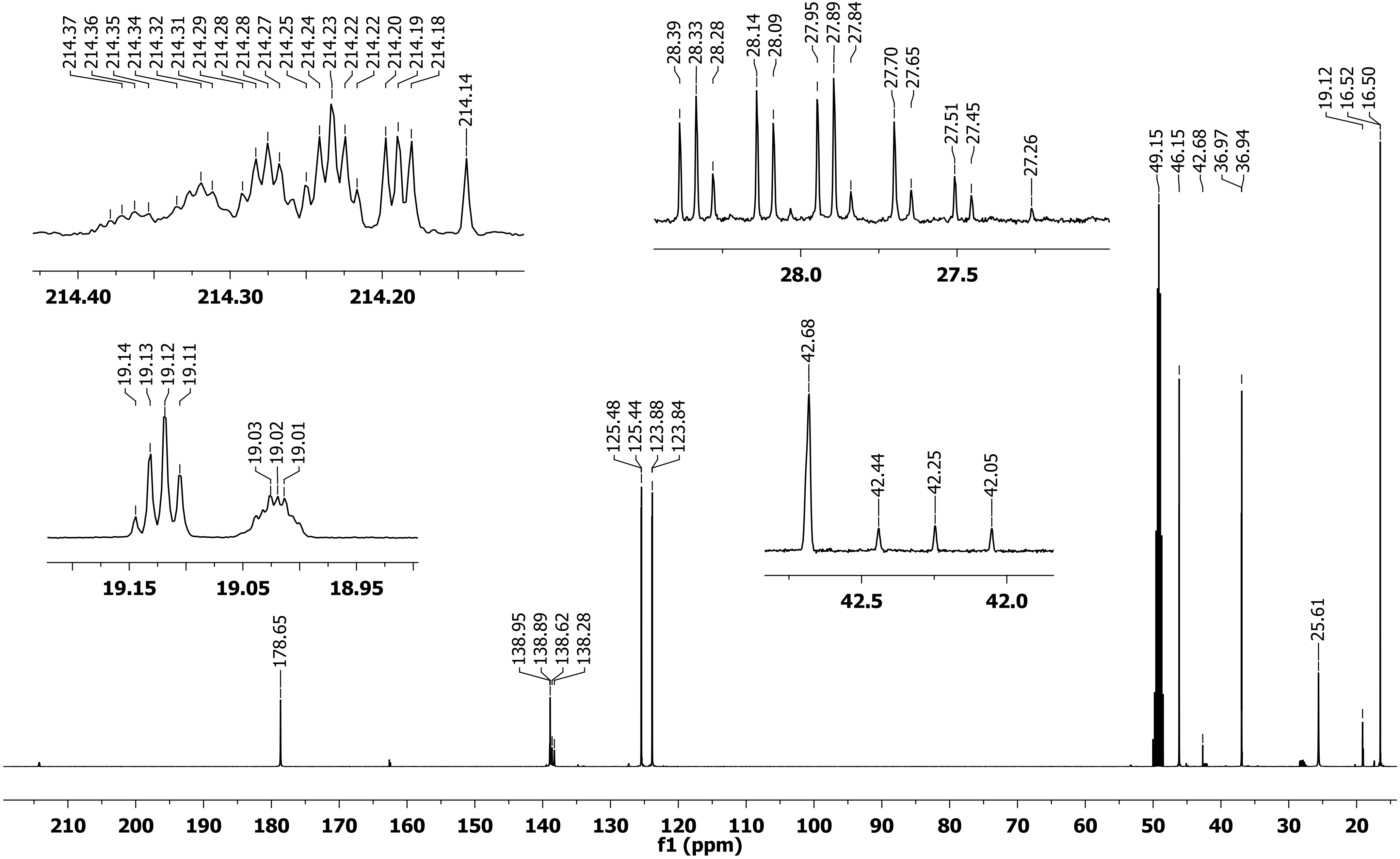

^{13}C NMR, Methyl ethyl ketone in $C_2mimOAc/CD_3OD$, after 1 day

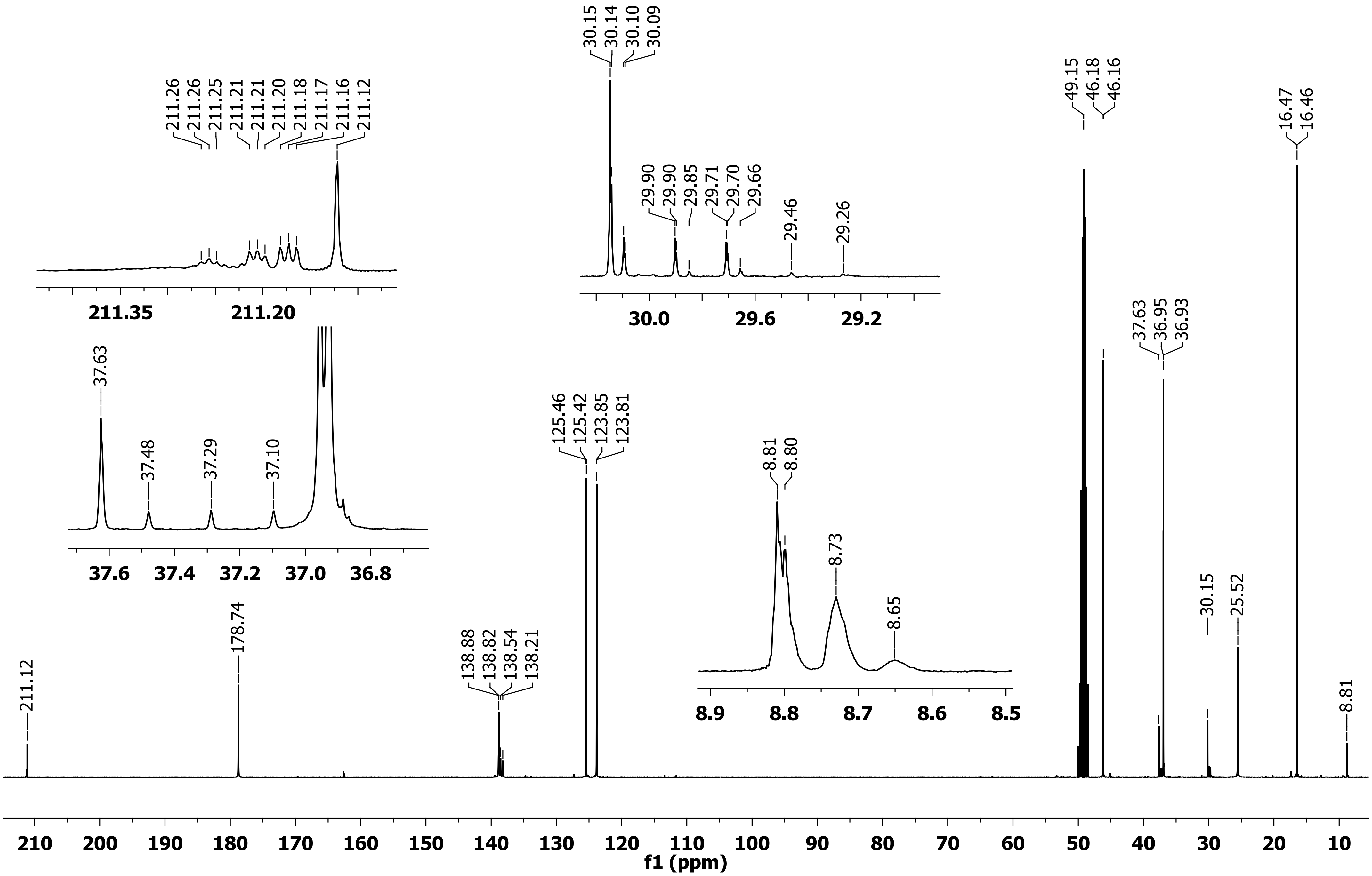

^{13}C NMR, Methyl ethyl ketone in $C_2mimOAc/CD_3OD$, after 2 days

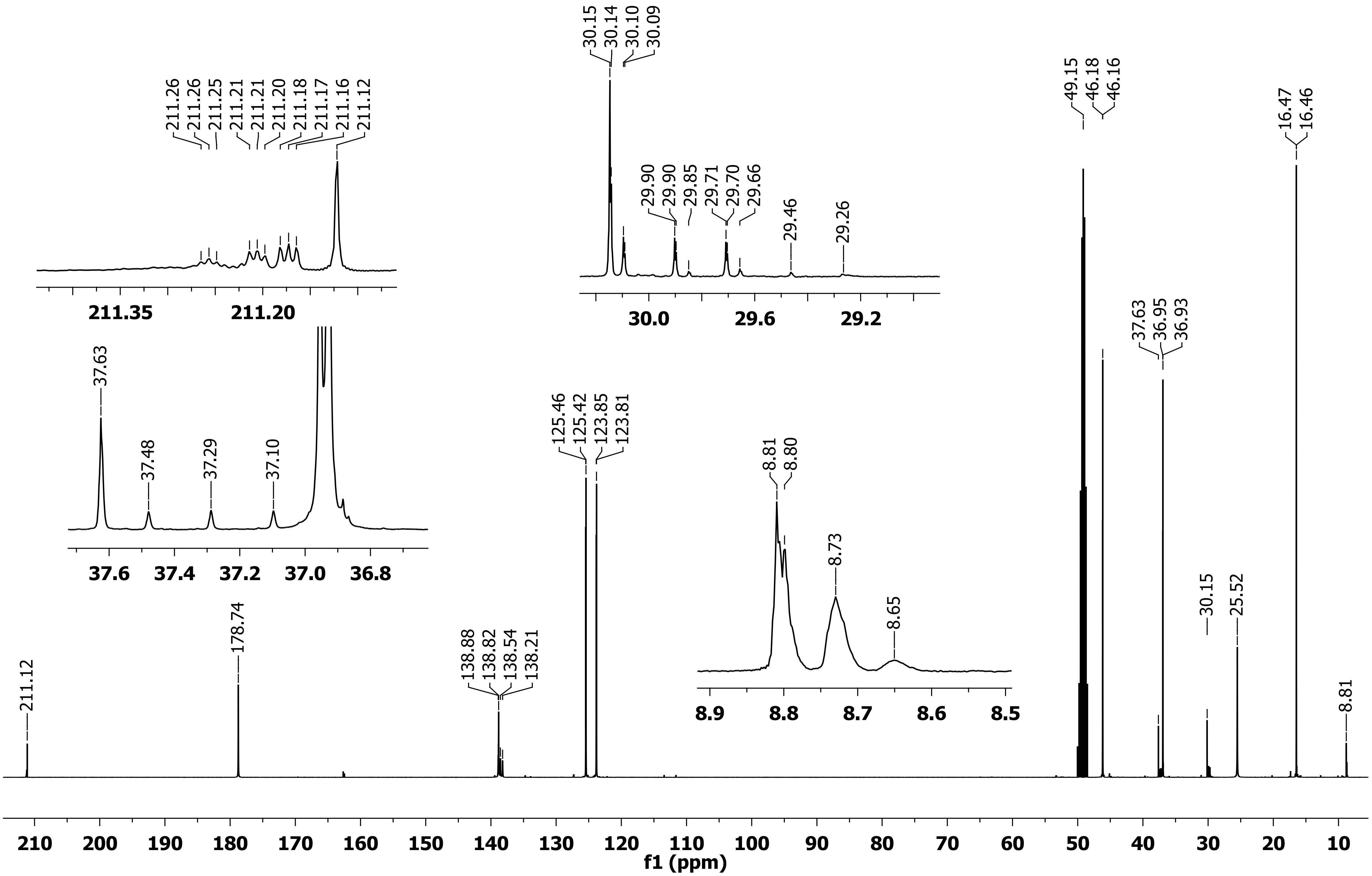

^{13}C NMR, Methyl ethyl ketone in $C_2mimOAc/CD_3OD$, after 11

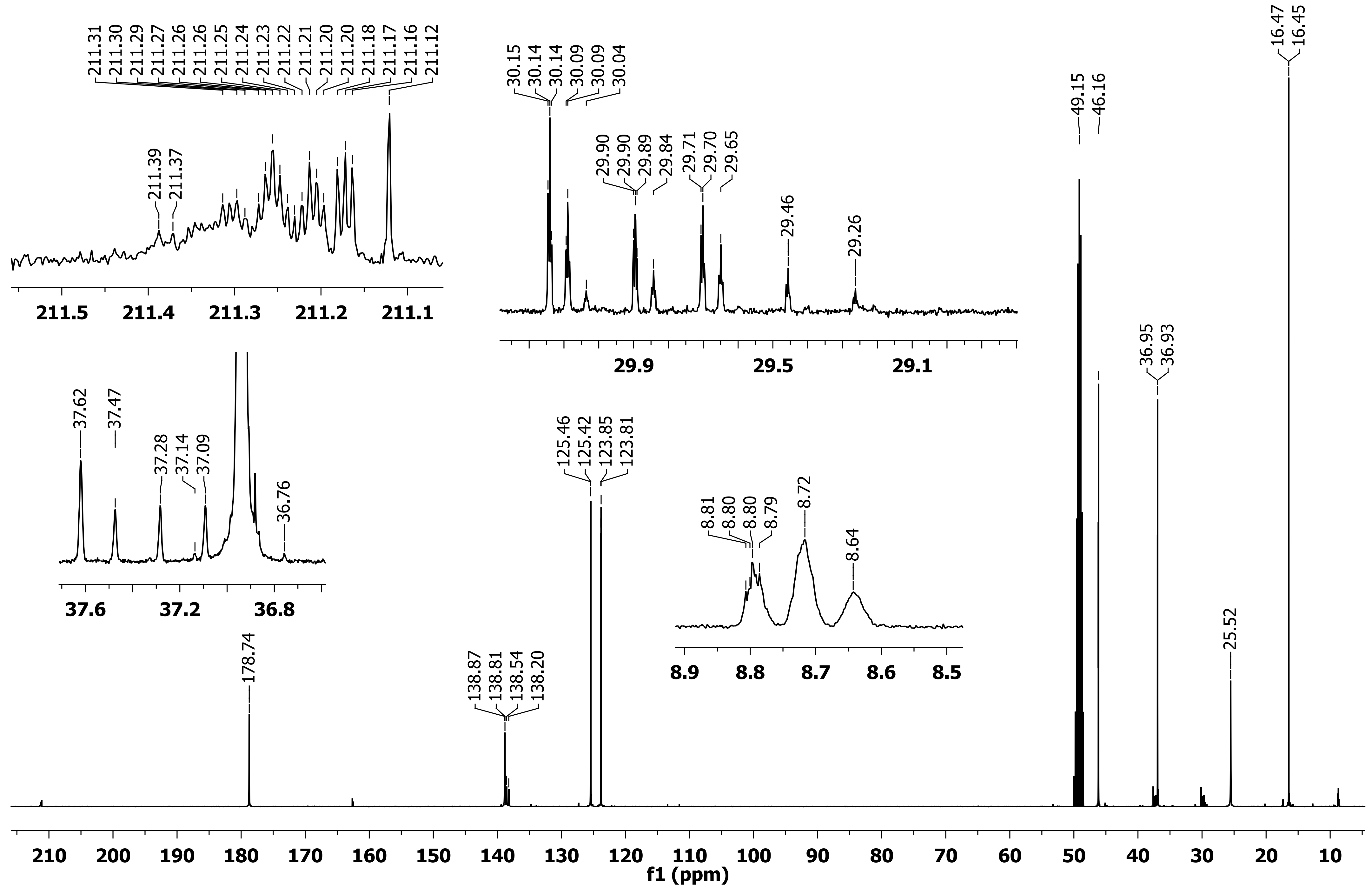

^{13}C NMR, Methyl ethyl ketone in $C_2mimOAc/CD_3OD$, after 6 weeks

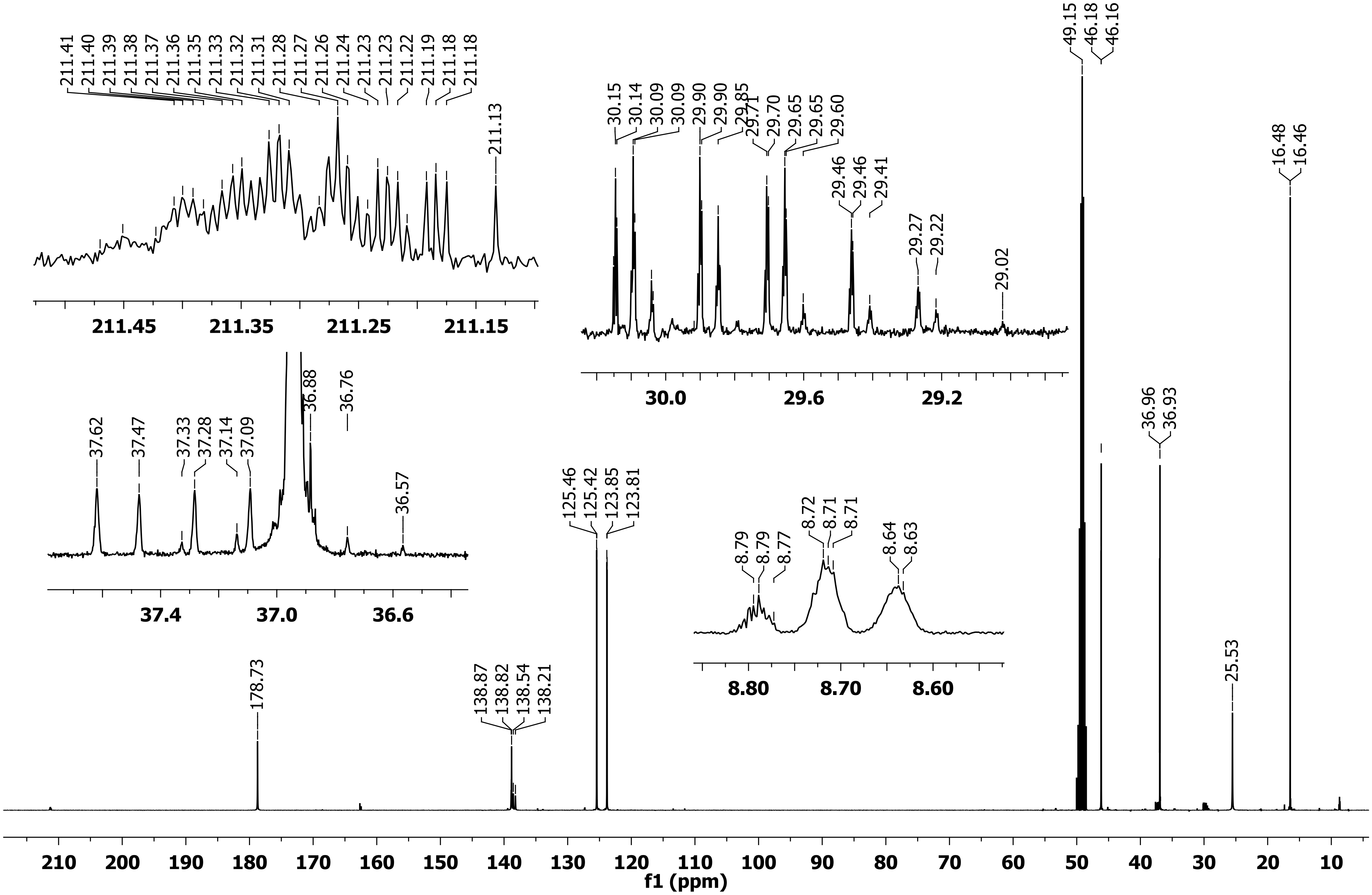

^{13}C NMR, Methyl Propyl Ketone in $C_2mimOAc/CD_3OD$, after 7 weeks

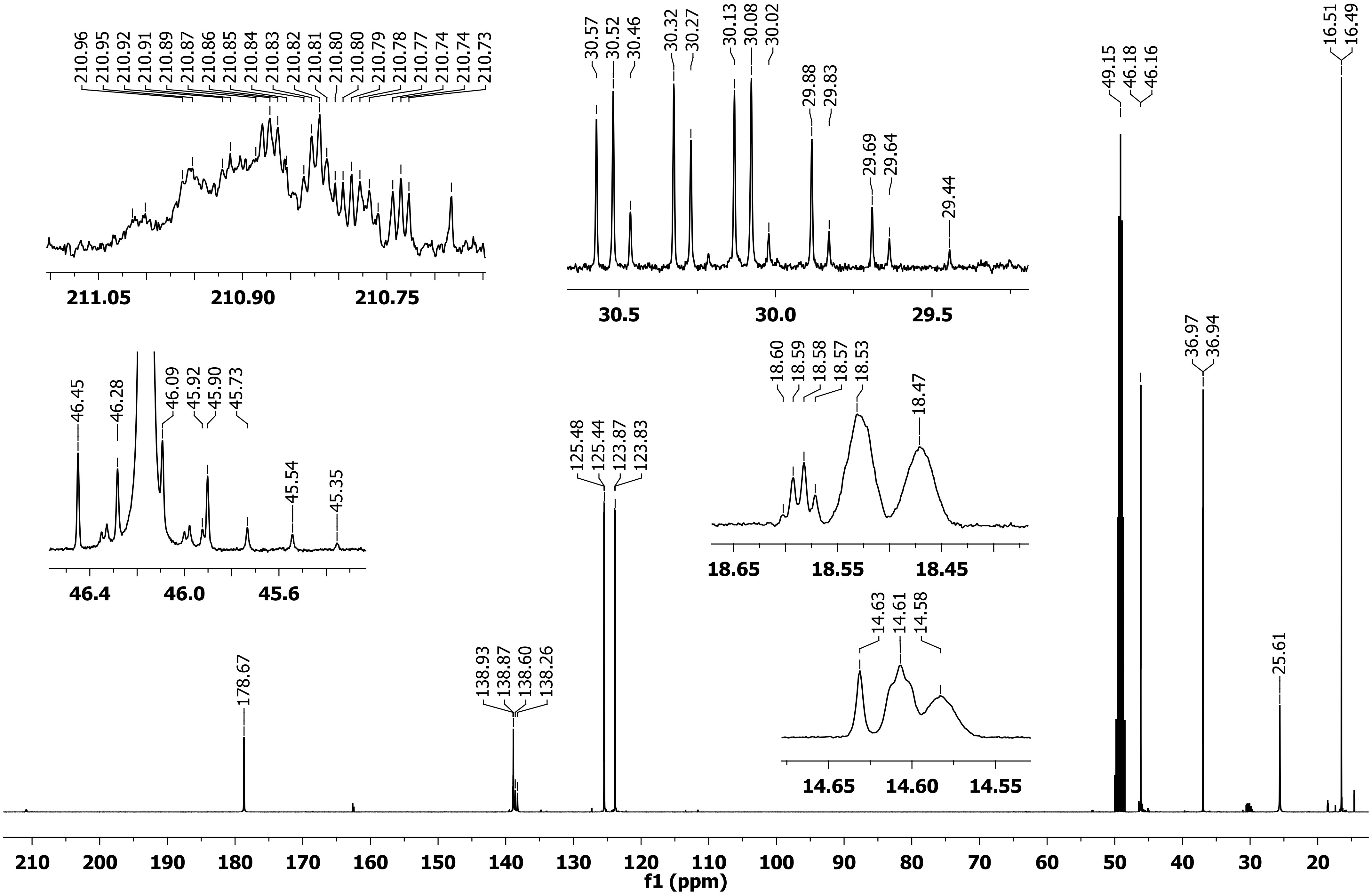

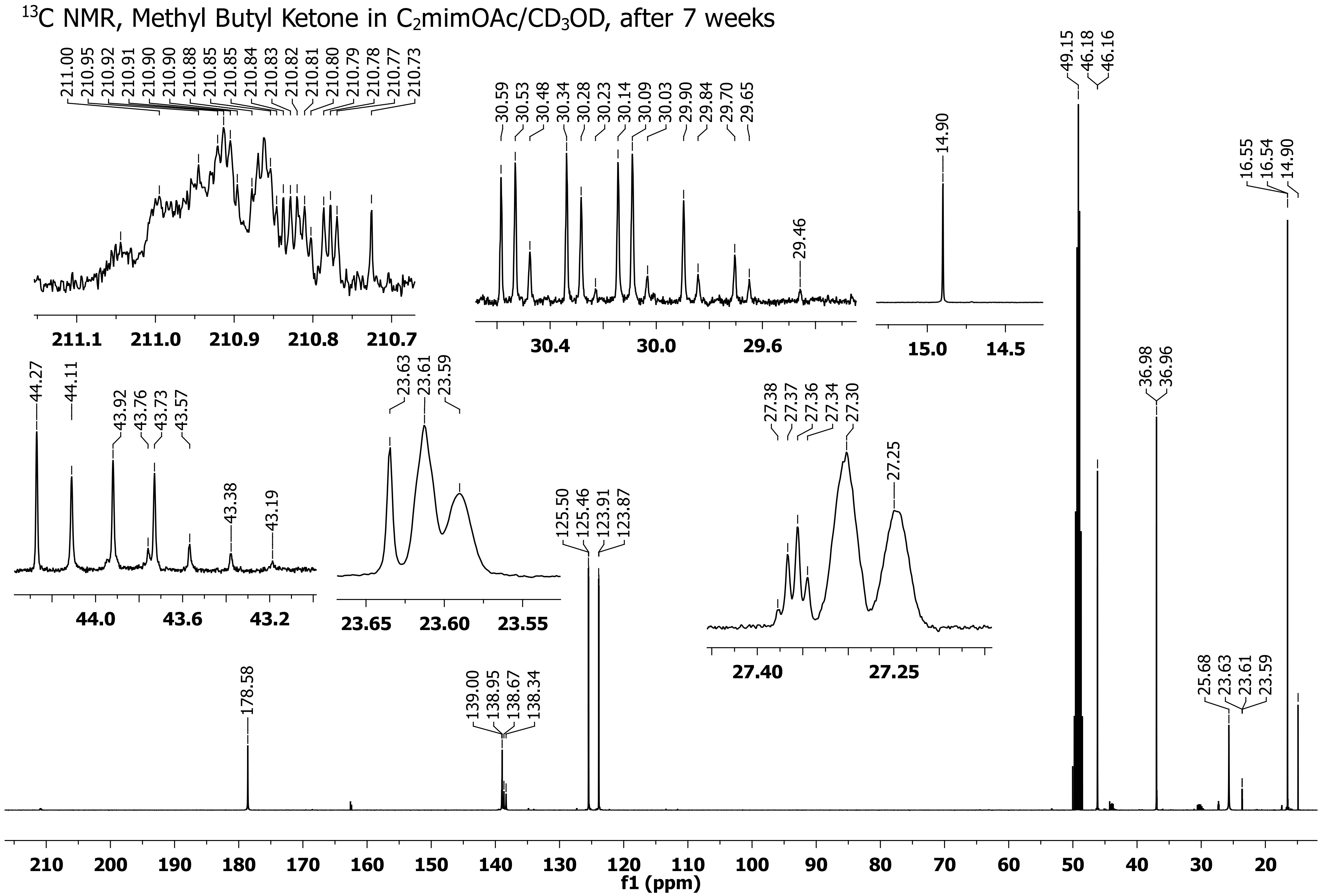

^{13}C NMR, Methyl Butyl Ketone in $C_2mimOAc/CD_3OD$, after 7 weeks
211.00 210.95 210.92 210.91 210.90 210.90 210.88 210.85 210.85 210.84 210.83 210.82 210.81 210.80 210.79 210.78 210.77 210.73
30.59 30.53 30.48 30.34 30.28 30.23 30.14 30.09 30.03 29.90 29.84 29.70 29.65
29.46
14.90
49.15 46.18 46.16
16.55 16.54 14.90
44.27 44.11 43.92 43.76 43.73 43.57
43.38 43.19
23.63 23.61 23.59
27.38 27.37 27.36 27.34 27.30
27.25
36.98 36.96
125.50 125.46 123.91 123.87
178.58
139.00 138.95 138.67 138.34
25.68 23.63 23.61 23.59
f1 (ppm)

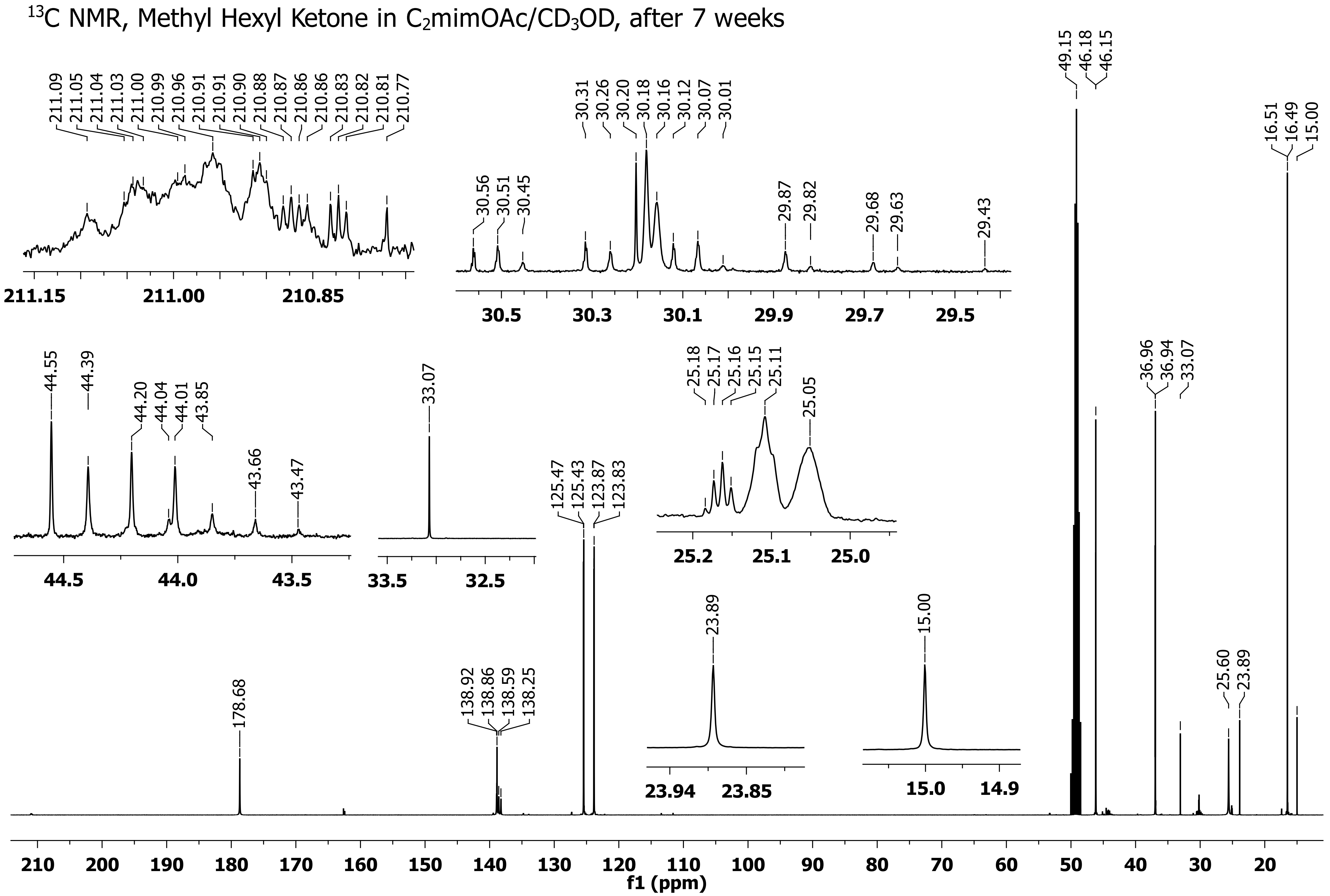

13C NMR, Methyl Hexyl Ketone in C2mimOAc/CD3OD, after 7 weeks
211.09
211.05
211.04
211.03
211.00
210.99
210.96
210.91
210.91
210.90
210.88
210.87
210.86
210.86
210.83
210.82
210.81
210.77
211.15
211.00
210.85
30.31
30.26
30.20
30.18
30.16
30.12
30.07
30.01
30.56
30.51
30.45
29.87
29.82
29.68
29.63
29.43
30.5
30.3
30.1
29.9
29.7
29.5
49.15
46.18
46.15
16.51
16.49
15.00
44.55
44.39
44.20
44.04
44.01
43.85
43.66
43.47
44.5
44.0
43.5
33.07
33.5
32.5
125.47
125.43
123.87
123.83
25.18
25.17
25.16
25.15
25.11
25.05
25.2
25.1
25.0
36.96
36.94
33.07
23.89
23.94
23.85
15.00
15.0
14.9
178.68
138.92
138.86
138.59
138.25
25.60
23.89
210
200
190
180
170
160
150
140
130
120
110
100
90
80
70
60
50
40
30
20
f1 (ppm)

^{13}C NMR, Methyl *Tert*-Butyl Ketone in $C_2mimOAc/CD_3OD$, after 3 weeks

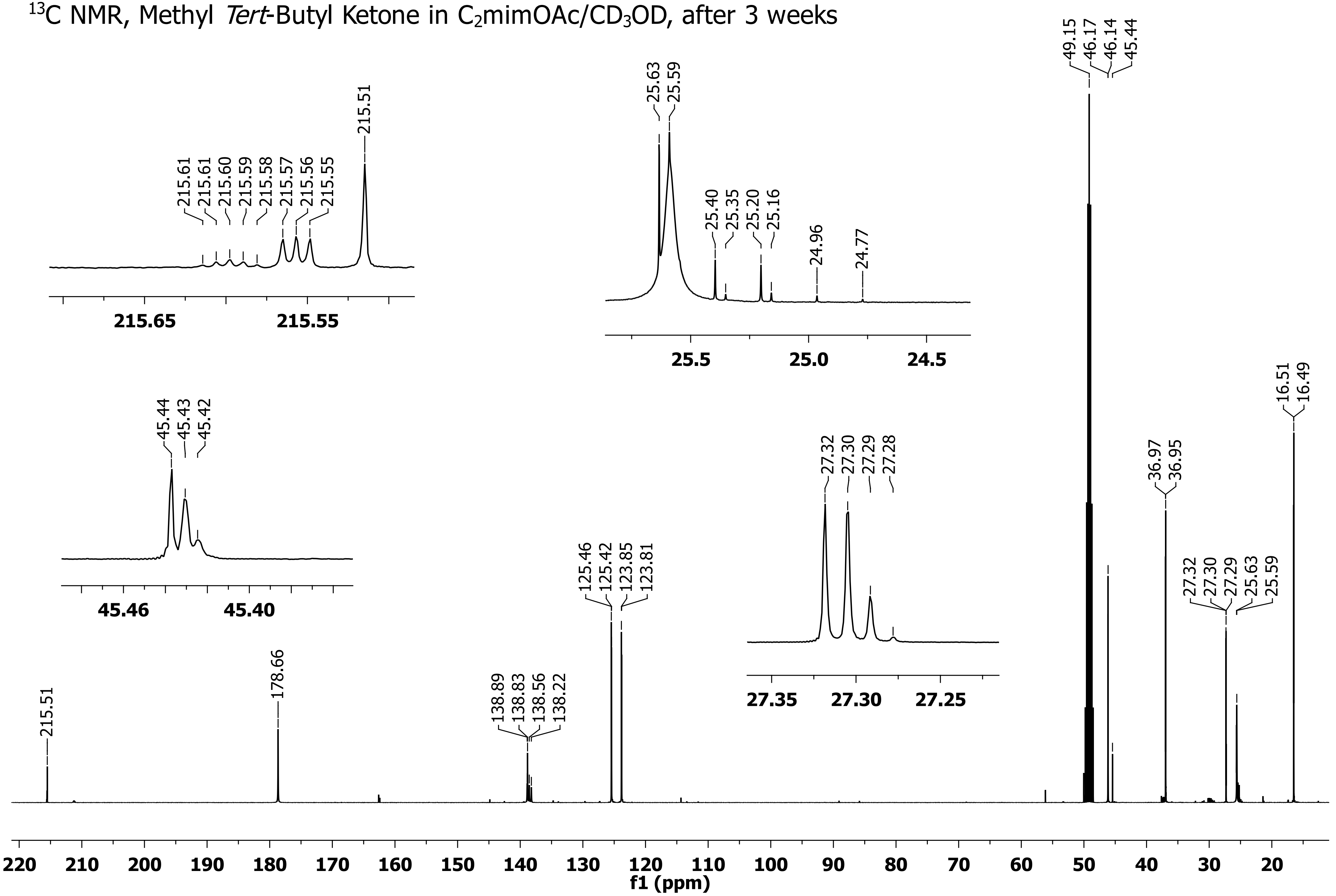

^{13}C NMR, Acetophenone in $C_2mimOAc/CD_3OD$, after 3 weeks

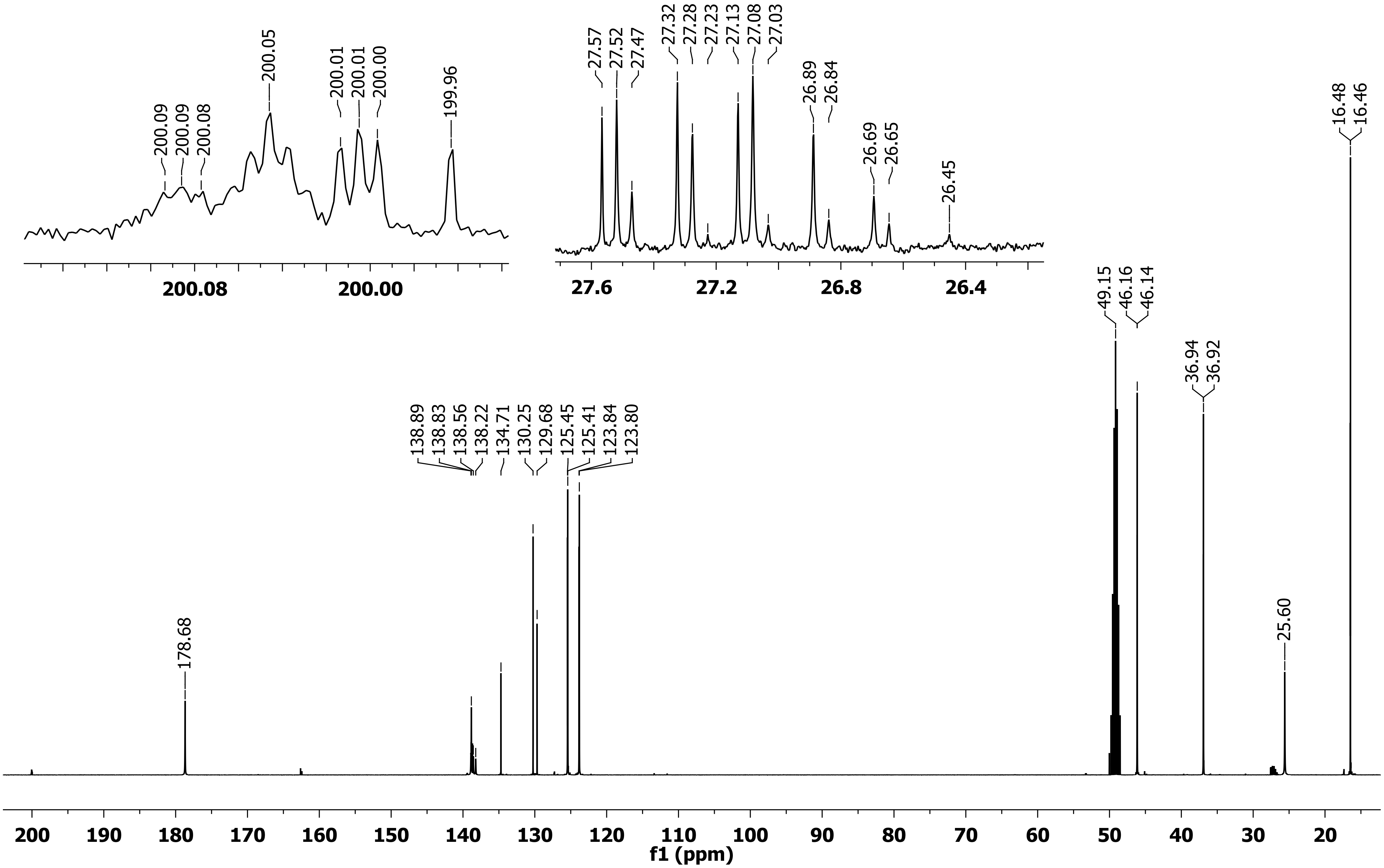

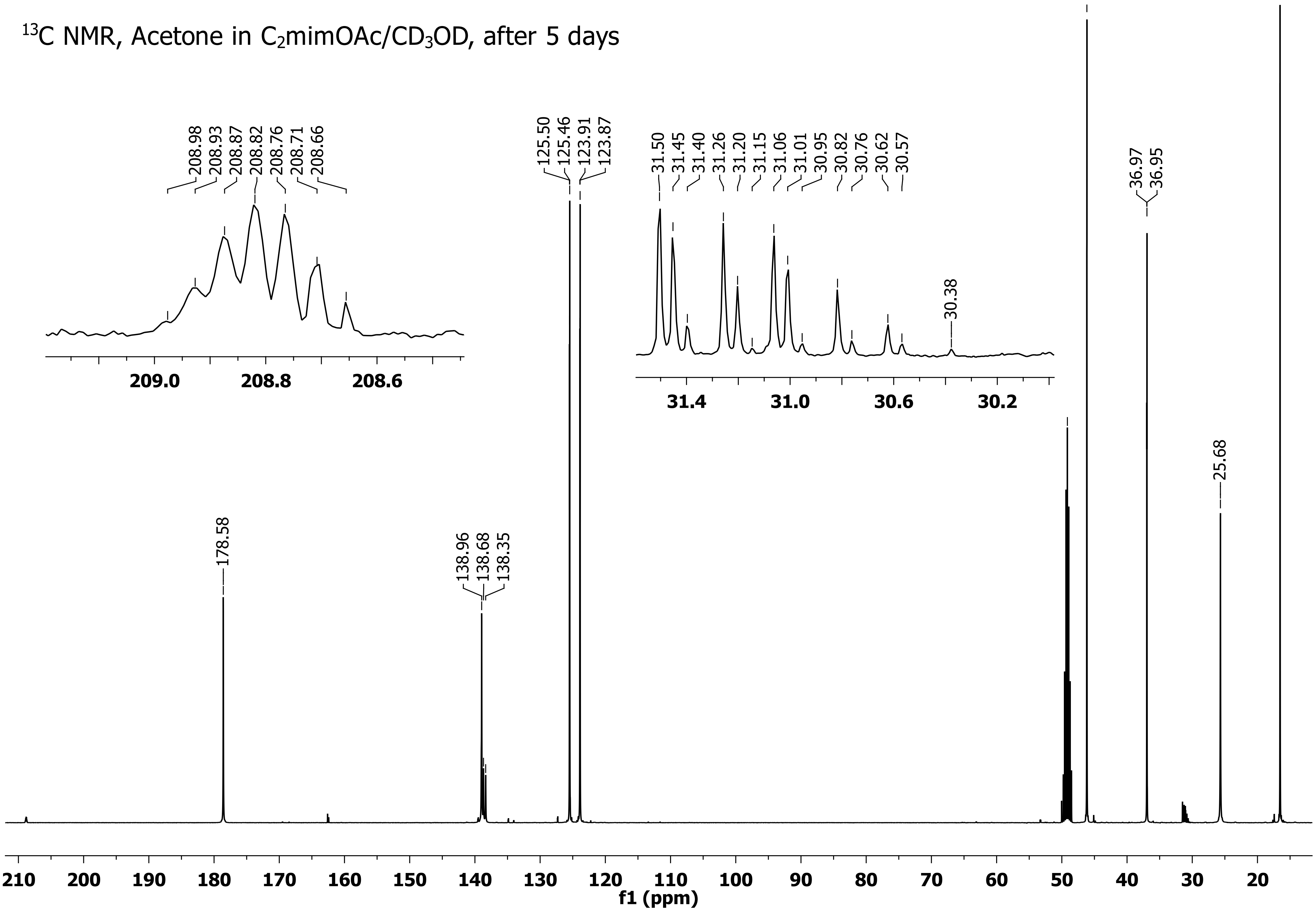

13C NMR, Acetone in C2mimOAc/CD3OD, after 5 days
208.98
208.93
208.87
208.82
208.76
208.71
208.66
209.0
208.8
208.6
125.50
125.46
123.91
123.87
31.50
31.45
31.40
31.26
31.20
31.15
31.06
31.01
30.95
30.82
30.76
30.62
30.57
30.38
31.4
31.0
30.6
30.2
36.97
36.95
25.68
178.58
138.96
138.68
138.35
210
200
190
180
170
160
150
140
130
120
110
100
90
80
70
60
50
40
30
20
f1 (ppm)

^1H NMR, Methyl Isopropyl Ketone in $C_2mimOAc/CD_3OD$, after 7 weeks

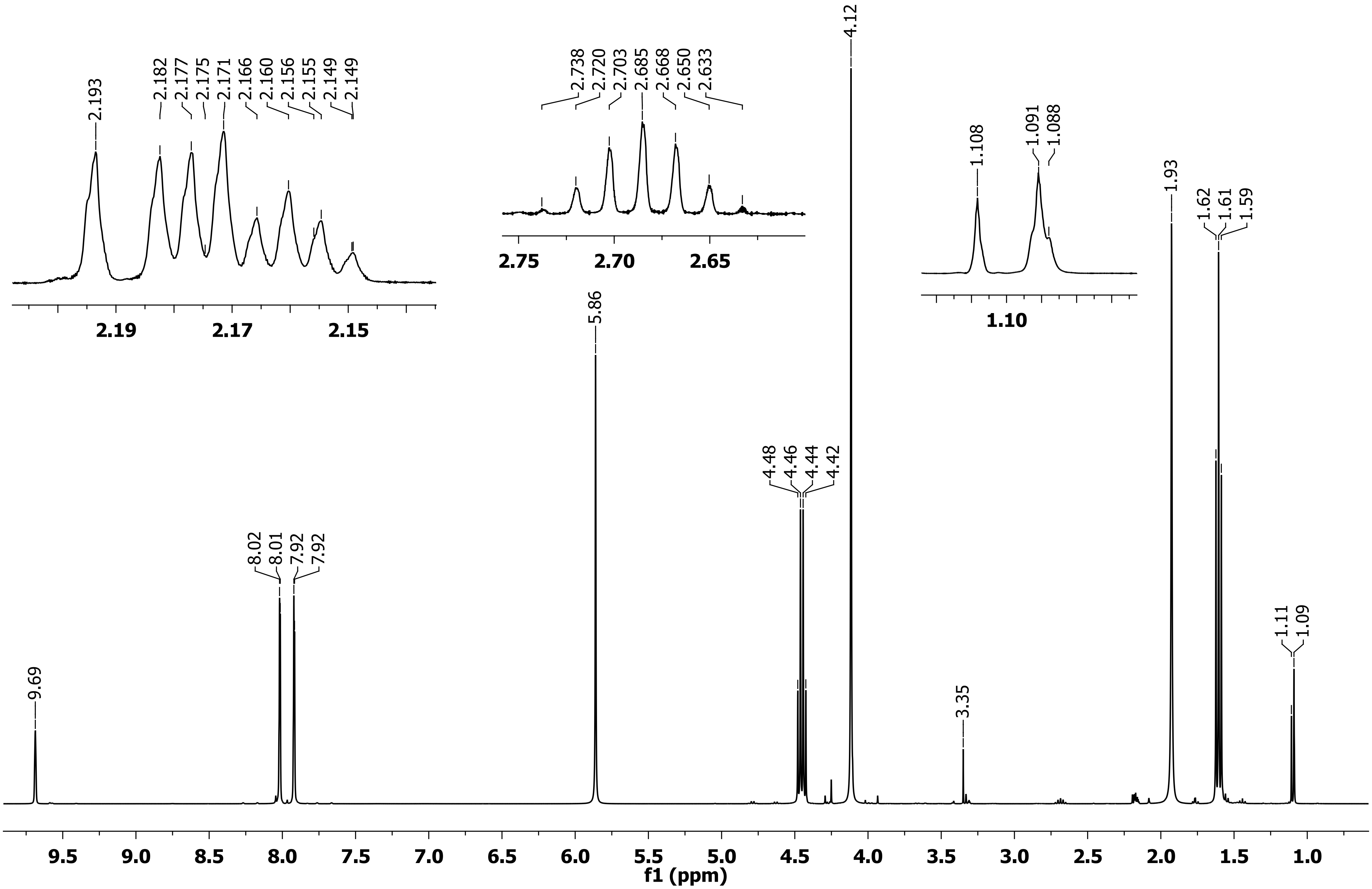

^1H NMR, Methyl Ethyl Ketone in $C_2mimOAc/CD_3OD$, after 7 weeks

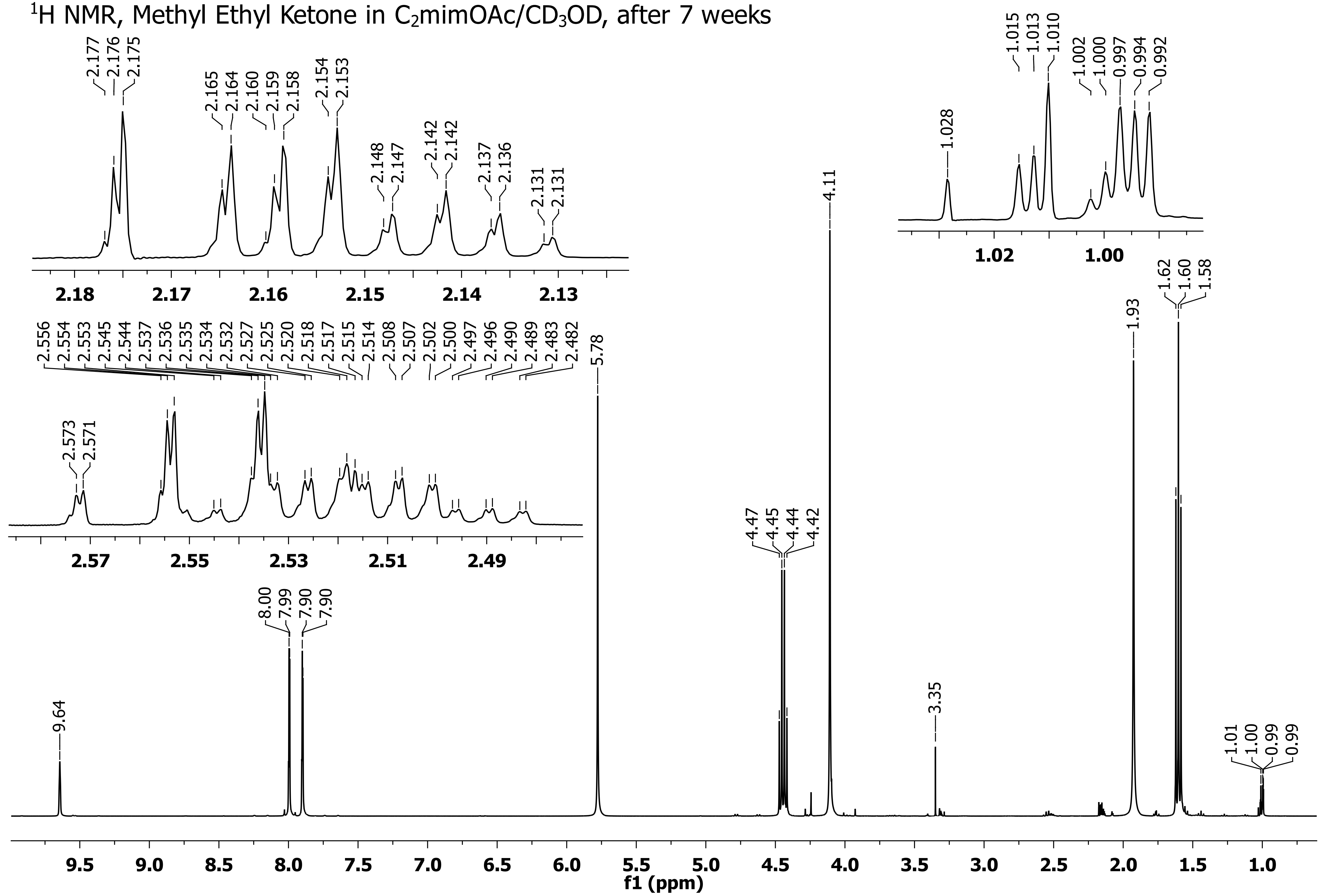

^{1}H NMR, Methyl Propyl Ketone in C_2mimOAc/CD_3OD, after 7 weeks

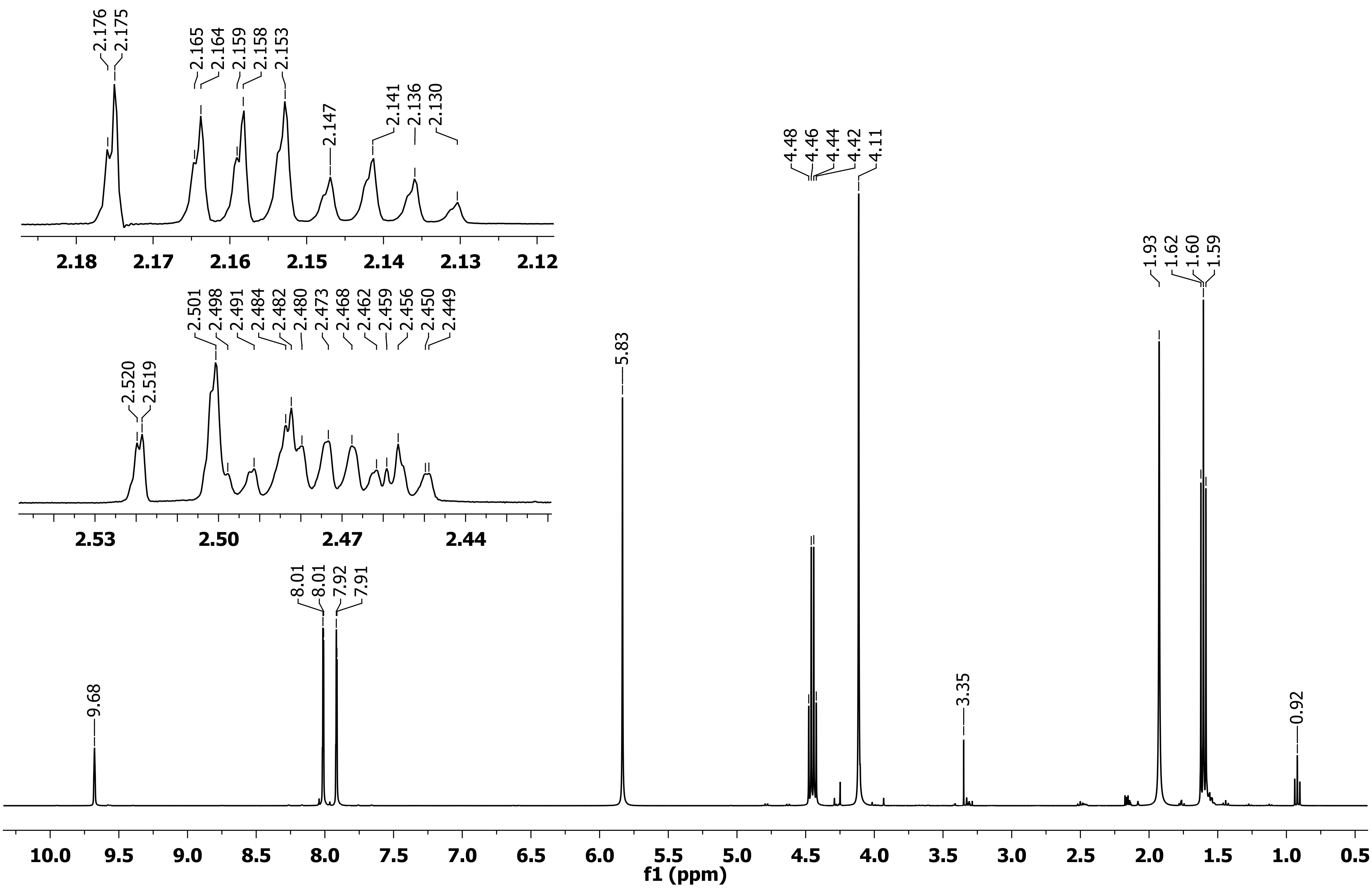

^1H NMR, Methyl Butyl Ketone in $C_2mimOAc/CD_3OD$, after 7 weeks

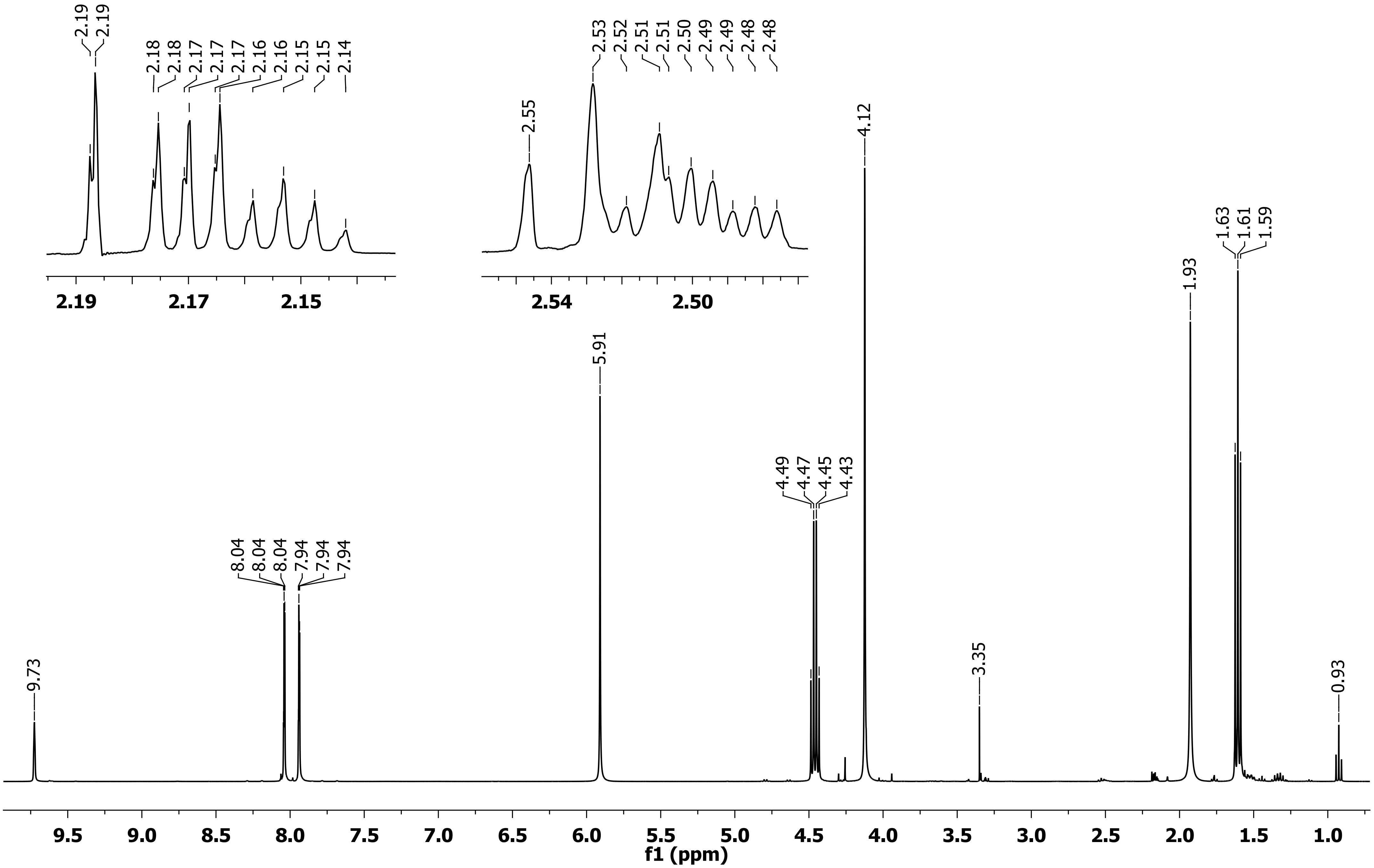

^{1}H NMR, Methyl Hexyl Ketone in C_2mimOAc/CD_3OD, after 7 weeks

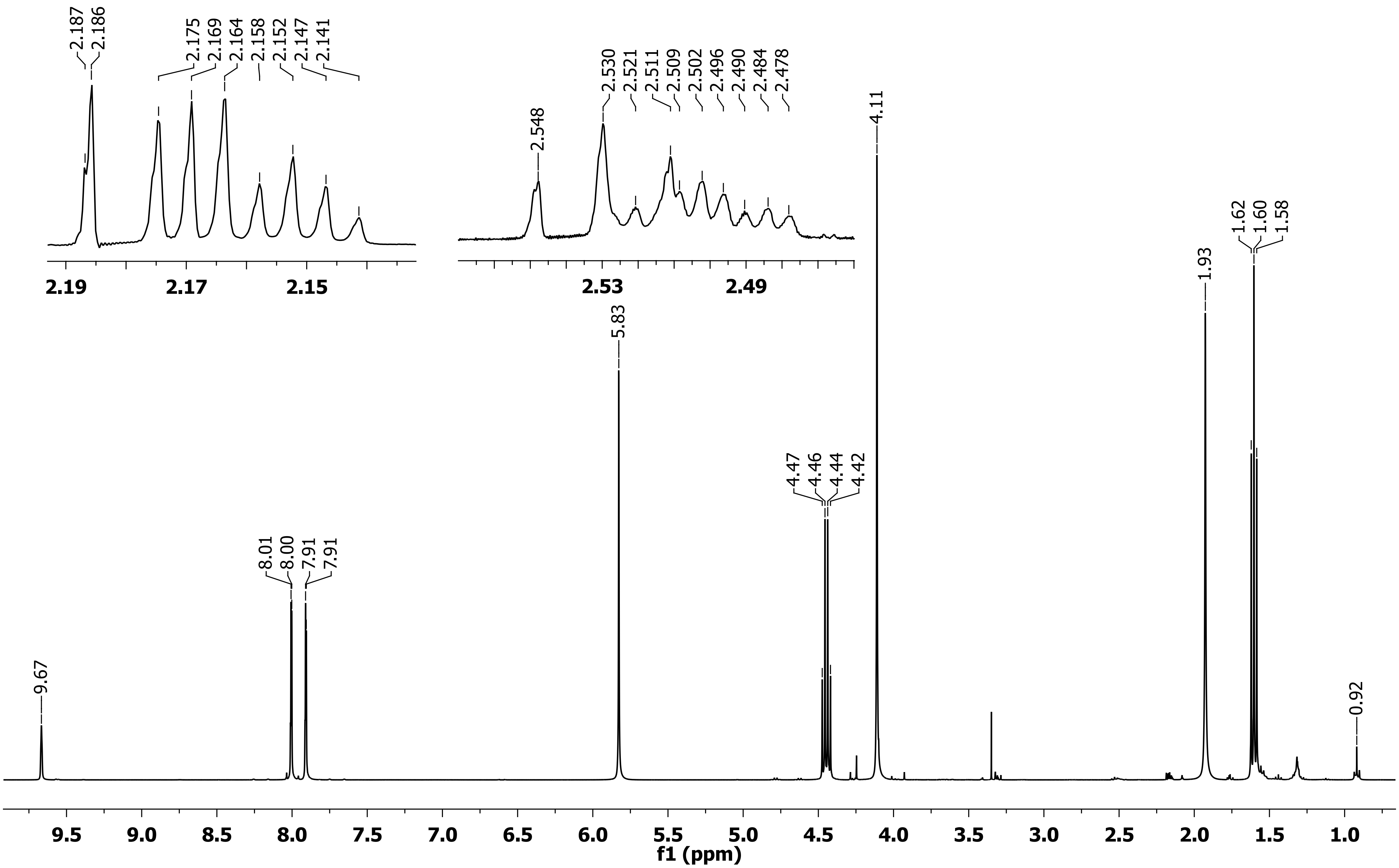

Supplement: RA-011-D1RA07232C-s001 [file RA-011-D1RA07232C-s001.pdf]
